# The Interplay of Weakly Coordinating Anions and the Mechanical Bond: A Systematic Study of the Explicit Influence of Counterions on the Properties of (Pseudo)rotaxanes

**DOI:** 10.3390/molecules28073077

**Published:** 2023-03-30

**Authors:** J. Felix Witte, Janos Wasternack, Shenquan Wei, Christoph A. Schalley, Beate Paulus

**Affiliations:** 1 Institut für Chemie und Biochemie, Physikalische und Theoretische Chemie, Freie Universität Berlin, Arnimallee 22, 14195 Berlin, Germany; 2 Institut für Chemie und Biochemie, Organische Chemie, Freie Universität Berlin, Arnimallee 20, 14195 Berlin, Germany

**Keywords:** computational chemistry, supramolecular chemistry, weakly coordinating anions, thermodynamics, energy decomposition analysis, isothermal calorimetry

## Abstract

Weakly coordinating anions (WCAs) have attracted much attention in recent years due to their ability to stabilise highly reactive cations. It may well be argued, however, that a profound understanding of what truly defines a WCA is still lacking, and systematic studies to unravel counterion effects are scarce. In this work, we investigate a supramolecular pseudorotaxane formation reaction, subject to a selection of anions, ranging from strongly to weakly coordinating, which not only aids in fostering our knowledge about anion coordination properties, but also provides valuable theoretical insight into the nature of the mechanical bond. We employ state-of-the-art DFT-based methods and tools, combined with isothermal calorimetry and ${}^{1}$H NMR experiments, to compute anion-dependent Gibbs free association energies $\Delta G_{a}$, as well as to evaluate intermolecular interactions. We find correlations between $\Delta G_{a}$ and the anions’ solvation energies, which are exploited to calculate physico-chemical reaction parameters in the context of coordinating anions. Furthermore, we show that the binding situation within the (pseudo)rotaxanes can be mostly understood by straight-forward electrostatic considerations. However, quantum-chemical effects such as dispersion and charge-transfer interactions become more and more relevant when WCAs are employed.

## 1. Introduction

While no charged species can ever truly be non-coordinating [1], research has proven that anions, e.g., as counterparts to highly reactive organic and inorganic cations [2,3,4], can be tailored in such a way that their coordination ability falls below that of the surrounding solvent. These species are referred to, quite descriptively, as weakly coordinating anions (WCAs) [5] and the search for the least coordinating one has been the focus of much research [6,7]. Fifty or so years ago, anions taking up slightly more space than mere halides, such as BF${}_{4}^{-}$ or ClO${}_{4}^{-}$, were considered to be non-coordinating, owing to a lack of experimental insight. Modern WCAs started to show up at the end of the last century, and frequently originate from the families of borates [8,9], aluminates [10,11,12,13], and carborates [14,15]. Applications of WCAs are plentiful, and range from catalysis [16,17] to organometallic chemistry [18] and supramolecular chemistry [19,20,21].

In spite of on-going investigations, it is fair to say that a comprehensive understanding of what constitutes a WCA is still lacking. Most modern examples contain a large perfluorinated shell, which aids in reducing coordination due to the low polarisability of the fluoride substituents. Systematic studies performed to assess the coordinative character of different anions are, however, scarce [22]. This study aimed to contribute to the fundamental research surrounding WCAs, by examining the influence of the coordination ability of a range of different anions on a supramolecular association reaction.

Charged species are frequently encountered in supramolecular systems, for example in host–guest complexes [23,24], or as external stimuli [25,26,27,28] for sensing or switching applications [29,30,31,32]. As strong binding is crucial for the assembly of molecular parts into supramolecules, WCAs are often a prerequisite for high yields. There are, however, cases in which the binding strength has to fulfill even stricter requirements. The functionality of switchable supramolecular systems, for example, would be disabled, if components bind too strongly to one another [33,34,35]. Fundamental knowledge about the role of environmental factors, such as counterions, is thus invaluable.

**Figure 1:**
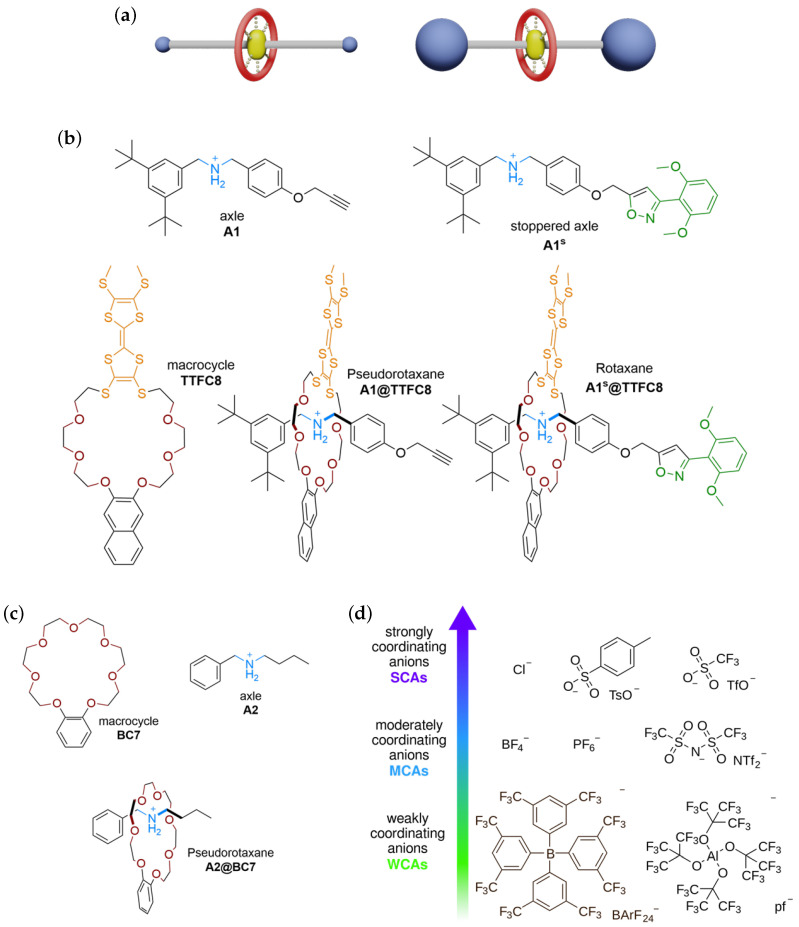
(**a**) Generic depiction of a pseudorotaxane (left) and a rotaxane (right) with non-covalent axle–wheel interactions indicated by dashed lines in the centre. (**b**,**c**) Supramolecular components investigated in this study. Note that the formation of **A1${}^{s}$@TTFC8** is not experimentally accessible, and was only examined on a theoretical basis. (**d**) Anions used in the reaction, categorised according to their potential ability to be weakly (WCA), moderately (MCA), or strongly (SCA) coordinating.

Rotaxanes and pseudorotaxanes (Figure 1a) are prominent examples of supramolecular structures. While the latter may readily convert back into their components (here, an ammonium axle and a crownether macrocycle), a mechanically interlocked rotaxane cannot dissociate. The interactions of the components with one another in a rotaxane may thus be considered as intra- rather than intermolecular. These species are of particular interest, as we may obtain insight into the nature of the mechanicals bond by comparing the experimentally accessible binding parameters of pseudorotaxanes with those of the corresponding interlocked rotaxanes. The direct formation of a rotaxane from its components is much more difficult to study experimentally, but can be analysed in detail by theory.

Supramolecular binding is governed by non-covalent interactions. The binding motifs in common pseudorotaxanes are often based on ion-dipole/ion-induced dipole and dispersion interactions, which formally decay with $R^{-4}$ and $R^{-6}$, respectively, with *R* being the inter-component distance. This is important, as the electrostatic contribution to the interaction of a positively charged axle with a negatively charged counterion depends on $R^{-1}$, representing a much larger range of effect. Even if binding is influenced by strong hydrogen bonds with a partially covalent character, the charge–charge interaction remains relevant. This underlines the importance of the choice of the counterion in studies involving pseudorotaxane formation.

In this work, we will investigate the thermodynamic and electronic properties of a supramolecular association reaction in the presence of various counterions, ranging from the strongly coordinating anions (SCAs) Cl${}^{-}$, F${}_{3}$CSO${}_{3}^{-}$ (OTf${}^{-}$), and *p*-tol-SO${}_{3}^{-}$ (OTs${}^{-}$), as well as the moderately coordinating anions (MCAs) BF${}_{4}^{-}$, PF${}_{6}^{-}$, and (F${}_{3}$CSO${}_{2}$)${}_{2}$N${}^{-}$ (NTf${}_{2}^{-}$), to the contemporary WCAs BArF${}_{24}^{-}$ and Al(OC(CF${}_{3}$)${}_{3}$)${}_{4}^{-}$ (pf${}^{-}$) (Figure 1b–d). Our main focus is on pseudorotaxane **A1@TTFC8**, which we will compare to its corresponding rotaxane **A1${}^{s}$@TTFC8**. To support our approach, a smaller and less complex pseudorotaxane **A2@BC7** was evaluated theoretically as well, details for which can be found in the ESI (Figure 1c and Section S6).

## 2. Results

In the following, we will utilise ITC and NMR experiments and literature values for benchmarking, as we employ state-of-the-art xTB [36] and DFT-based methods to examine molecular structures and Gibbs free energies of association $\Delta G_{a}$. Our computational results suggest a correlation between $\Delta G_{a}$ and solvation free energies $\Delta G_{\mathrm{solv}}$, which is exploited to define reaction parameters used to describe the supramolecular reaction at hand, in the context of coordinating anions. In addition, we perform energy decomposition analyses, based on the fragment molecular orbital method (FMO-EDA) [37,38], in order to assess inter-component interactions. Decomposing the binding energies displays the significance of electrostatic and dispersion contributions to the total interaction, providing valuable theoretical insight into the nature of the mechanical bond.

### 2.1. Pseudorotaxane Formation in ITC and NMR Experiments

ITC experiments provide a means to obtain thermodynamic data, such as changes in enthalpy ΔH and entropy ΔS, for reactions of non-covalently interacting species. The measurements reveal a trend for the Gibbs free association energy $\Delta G_{a}$ of the pseudorotaxane axle **A1** and the macrocycle wheel **TTFC8,** in agreement with chemical intuition, as one might argue (Table 1). The strong and weak coordination properties of the anions are clearly reflected within this trend. For SCAs Cl${}^{-}$ and OTf${}^{-}$, ${}^{1}$H NMR experiments rule out any significant binding of **TTFC8** and **A1**. This observation is in agreement with the literature [39,40], and can be explained by the strong interaction of the anion with the ammonium axle, as we will see further down below. At the weakly coordinating end of the spectrum of anions, a plateau is reached: **A1** binds at the same strength, irrespective of BArF${}_{24}^{-}$ or pf${}^{-}$ being present as the counterion.

While modern experimental techniques are extremely versatile tools, used to understand intermolecular bonding and non-covalent interactions, there are a few limitations. For example, the barrier for rotaxane formation from the free intact axle and wheel is too high to directly measure the Gibbs free association energies. Moreover, since we are interested in the mechanical bond, we would like to inquire as to how different contributions, such as dispersion or electrostatics, may contribute to the intramolecular inter-component binding within a rotaxane, with and without specific counterions present. This is, again, something we cannot directly deduce from experimental thermodynamic values, as they always represent a macroscopic sum of these contributions.

Theoretical methods, on the other hand, do not suffer from these limitations. A $\Delta G_{a}$ value for the hypothetical formation of a rotaxane from its components is readily accessible from standard approaches. Furthermore, a more detailed understanding of intramolecular interactions can be accomplished through methods based on an energy-decomposition ansatz [38,45,46]. Of course, quantum–chemical theory also has several caveats, for example, when solvent molecules strongly interact with a solute or—perhaps the most noticeable drawback—when there is a necessity for approximations of large molecules [47,48,49]. Hence, herein, we use a combined approach and benchmark our theoretical calculations to our ITC measurements. More details regarding benchmarking calculations can be found in the ESI (Table S1).

In the remaining sections, we will explore the thermodynamic and electronic aspects of the pseudorotaxane and hypothetical rotaxane formation reactions. This entails an analysis of conformational landscapes and a detailed evaluation of the interactions between the axle, wheel, and counterions in both the pseudorotaxane and the rotaxane.

### 2.2. Pseudorotaxane Formation In-Silico

Benchmarking was conducted for three different density functions, PBE0-D3 (BJ) [50,51,52], M06-2X [42], and ωB97X-D3 [53], all of which should provide accurate thermochemical data for organic reactions [54,55]. Here, $\Delta G_{a}$ is computed according to a re-structured version of the Gibbs–Helmholtz equation,

(1) $$\Delta G_{a}=\Delta E_{\mathrm{el}}+\Delta G_{\mathrm{therm}}$$

where inputs are directly obtainable from common quantum–chemistry codes. $\Delta E_{\mathrm{el}}$ is the difference in electronic energies and $\Delta G_{\mathrm{therm}}$ is the thermal correction term, containing enthalpic and entropic contributions, which are calculated from normal modes employing the rigid rotor harmonic oscillator approximation [56], as well as the single-point Hessian approach [57] by Grimme and co-workers.

The M06-2X method in combination with the SMD solvent model [41] yields results that are in remarkable agreement with experiments on most association reactions. Complexes with Cl${}^{-}$ and especially BF${}_{4}^{-}$ are overstabilised, which is likely caused by the absence of explicit solvent molecules in the calculations. Due to their small sizes, these anions need more directly coordinating solvent molecules to compensate their localised charge. This is probably true for PF${}_{6}^{-}$ as well, which, however, yields a $\Delta G_{a}$ value that is close to experimental values, possibly due to fortuitous error cancellation. To ensure consistency, however, no explicit solvent molecules were included in any system, as obtaining converged results with respect to the number of solvent molecules is quite cumbersome and beyond the scope of this paper.

**Figure 2:**
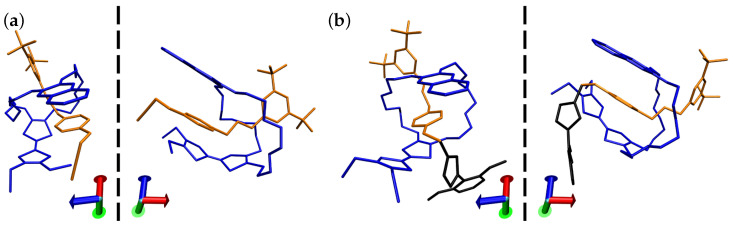
Most stable co-conformations of (**a**) **A1@TTFC8** and (**b**) **A1${}^{s}$@TTFC8**, each shown from two perspectives without counterions. The axle, macrocycle, and stoppering unit are depicted in orange, blue, and black, respectively. Hydrogen atoms are omitted for clarity. Axes are added for convenience.

To obtain reliable theoretical results, a workflow was first established, starting with a scan of the conformational landscape of each cation-anion combination. All anions, not just SCAs such as Cl${}^{-}$ or OTf${}^{-}$, exert an apparent influence, resulting in a relatively shallow potential energy surface (PES), and a plethora of different (co-)conformers for ammonium axles, pseudorotaxanes, and rotaxanes (Figure S15). However, our calculations suggest that the most stable pseudorotaxane and rotaxane co-conformations are always retained, irrespective of which anion is present (Figure 2). This is not only the case for **A1@TTFC8** and **A1${}^{s}$@TTFC8**, but also for **A2@BC7** (Figure S20).

The most stable co-conformations of **A1@TTFC8** and **A1${}^{s}$@TTFC8** can essentially be described as the same kind of densely packed structure, differing only in the existence of the stoppering unit in the latter. Due to the strong inter-component interactions within **A1@TTFC8**, neither extension by an isoxazole unit to form **A1${}^{s}$@TTFC8** nor any of the counterions are able to interfere with its conformational stability, although for some counterions, the conformational diversity is clearly increased. In addition, the counterion seems to remain around the same position, in an almost-stable conformation. For **A1@TTFC8**, this is close to the crown ether hydrogen atoms and one of the SMe groups of the TTF moiety (Figure S16). Other positions appear to be disfavoured (Figure S17).

The observed structural consistency is quite intriguing, as this is in no way congruent with the computed $\Delta G_{a}$ values, for which the different anions have a significant effect. Interestingly, while axles **A1**, **A1${}^{s}$**, and **A2** are each stabilised through their interactions with nearly all the anions, with very few exceptions, the pseudorotaxanes **A1@TTFC8** and **A2@BC7** and rotaxane **A1${}^{s}$@TTFC8** are stabilised by none of them (Tables S2–S4). This is mostly due to the electronic energy $\Delta E_{\mathrm{el}}$, as differences in $\Delta G_{\mathrm{therm}}$ are practically negligible, usually below 5 kJ/mol (Table S5). In this way, we may thus regard **A1@TTFC8**, **A1${}^{s}$@TTFC8**, and **A2@BC7** as weakly coordinating cations.

As expressed earlier, a potent WCA should not interact with reactants and product with greater efficacy than the surrounding solvent molecules. This made us wonder, whether we could exploit the solvation free energies $\Delta G_{\mathrm{solv}}$ of the anions and their relationships with $\Delta G_{a}$, in order to gain useful information about their coordination properties. Here, we have computed $\Delta G_{\mathrm{solv}}$ simply as the difference in Gibbs free energies between the anion in vacuum and in solution, as described by the SMD [41] implicit solvent model,

(2) $$\Delta G_{\mathrm{solv}}=G^{\mathrm{SMD}}-G^{\mathrm{vac}}.$$

However, other approaches, such as COSMO-RS [58], may be employed as well. For all physically sound-charged systems $\Delta G_{\mathrm{solv}}$, will be negative. As with most natural processes, it makes sense to assume an exponential function to connect $\Delta G_{\mathrm{solv}}$ and $\Delta G_{a}$,

(3) $$\frac{\left(\Delta G_{a}-\Delta G_{a}^{\mathrm{opt}}\right)}{RT}=\frac{\Delta \Delta G_{a}}{RT}=k_{1}\exp\left(-k_{2}\frac{\Delta G_{\mathrm{solv}}}{RT}\right).$$

$\Delta G_{a}^{\mathrm{opt}}$ is the extrapolated association free energy in the limit of the perfectly non-coordinating anion, and $k_{1}$ and $k_{2}$ can be interpreted as reaction-dependent coordination constants. Equation (3) is linearised and iteratively sampled using different $\Delta G_{a}^{\mathrm{opt}}$ values, until convergence of the determination coefficients is observed. The extracted parameters are shown in Table 2.

Unfortunately, both $\Delta G_{a}$ and $\Delta G_{\mathrm{solv}}$ are rather error-prone in terms of Cl${}^{-}$, BF${}_{4}^{-}$, and PF${}_{6}^{-}$, as discussed before. It may, thus, be argued that these anions should be omitted from the analysis. Doing so curiously yields a remarkably distinct linear trend for the logarithmic plot of $\Delta G_{a}$ against $\Delta G_{\mathrm{solv}}$, with determination coefficients close to unity (Figure S18). We note, however, that the validity of this trend is somewhat debatable, as there are only five data points.

Interestingly, a $\Delta G_{a}$ of −49.0 kJ/mol is found for **A1@TTFC8** when no counterion is present, which is significantly lower than the −33.8 kJ/mol obtained for $\Delta G_{a}^{\mathrm{opt}}$(**A1@TTFC8**). This notable difference underlines the importance of regarding explicit counterion effects in calculations. Larger uncertainties are frequently encountered for charged molecular systems in solution [54,59,60].

Furthermore, it may be argued that the reaction parameter $k_{2}$ carries more information than $k_{1}$, as the former has a much more dramatic effect on the shape of the exponential function. To extract meaningful information from these parameters, however, the counterion effects of other types of reactions will have to be studied. Nevertheless, we tentatively propose that $\Delta G_{\mathrm{solv}}$, and, thus, $k_{1}$ and $k_{2}$, can be used as a measure of the coordinative character of anions for a specific reaction.

### 2.3. Inter-Component Interactions in Pseudorotaxanes and Rotaxanes

What are the changes within a supramolecule-like **A1@TTFC8**, when the intermolecular interactions turn intramolecular upon the formation of **A1${}^{s}$@TTFC8**? To gain a more fundamental understanding of the inter-component interactions in our molecules, we employed analytical quantum–chemical approaches.

**Figure 3:**
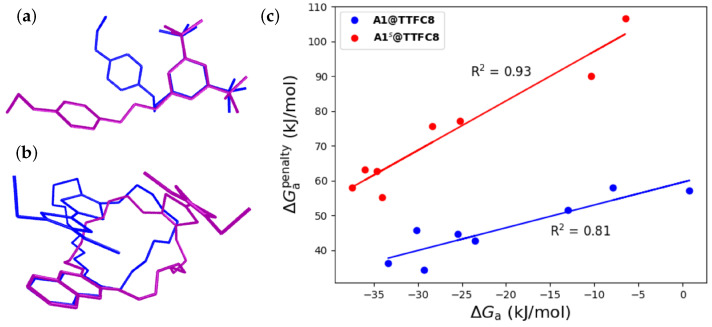
Illustration of the structural penalty, using the example of (**a**) **A1** without a counterion and (**b**) **TTFC8**; the structures in blue correspond to the free structure, while the purple ones represent the structure in the supramolecule. (**c**) Plot of $\Delta G_{a}$${}^{\mathrm{penalty}}$ vs. $\Delta G_{a}$ for **A1@TTFC8** (blue) and **A1${}^{s}$@TTFC8** (red). The coefficients of determination are given for convenience.

To relate Gibbs free association energies, inter-component interactions, and counterion influence, we evaluated the structural penalty of the formation reaction $\Delta G_{a}$${}^{\mathrm{penalty}}$ = $\Delta G^{\mathrm{penalty}}$(**A1**) + $\Delta G^{\mathrm{penalty}}$(**TTFC8**). $\Delta G^{\mathrm{penalty}}$ describes the Gibbs free energy associated with forcing one of the components into its structure within the supramolecular system. $\Delta G_{a}$${}^{\mathrm{penalty}}$ can become quite large (up to 100 kJ/mol), and correlates nicely with computed $\Delta G_{a}$ values for **A1@TTFC8**, and especially for **A1${}^{s}$@TTFC8** (Figure 3 and Tables S6 and S7). Note that the single-point Hessian approach employed for the evaluation of the $\Delta G_{\mathrm{therm}}$ contributions to $\Delta G_{a}$${}^{\mathrm{penalty}}$ compensates for the fact that the free energies from normal mode analyses are somewhat ill-defined for non-equilibrium structures.

The structural penalties for **A1${}^{s}$@TTFC8** are consistently larger than for **A1@TTFC8**. The main contribution here is the destabilisation of the macrocycle, which is, on average, roughly 20 kJ/mol higher for **A1${}^{s}$@TTFC8** than for **A1@TTFC8** compared to the destabilisation of the ammonium axle, with a difference of ca. 7 kJ/mol, on average. The slope in Figure 3c, on the other hand, is clearly related to the properties of the involved anions, with SCAs causing almost twice as much of a penalty as WCAs. This effect is slightly more pronounced for **A1${}^{s}$@TTFC8**, which is likely due to the larger size of **A1${}^{s}$**, and makes larger changes necessary for it to be incorporated in **A1${}^{s}$@TTFC8**. Despite the higher structural penalty for **A1${}^{s}$@TTFC8** in comparison to **A1@TTFC8**, its $\Delta G_{a}$ value is slightly lower, indicating stronger inter-component interactions, which overcompensate the $\Delta G_{a}$${}^{\mathrm{penalty}}$ in **A1${}^{s}$@TTFC8** more than in **A1@TTFC8**.

Energy decomposition analysis based on the fragment molecular orbital method (FMO-EDA) [37,38] can be used to obtain a qualitative idea of the inter-component interactions [61] in **A1@TTFC8** and **A1${}^{s}$@TTFC8**, thus, providing insight into the mechanical bonds. Stronger dispersion interactions $E_{\mathrm{Disp}}$ upon rotaxane formation are almost fully counterbalanced by an increase in exchange interactions $E_{X}$, while the electrostatic contribution $E_{\mathrm{ES}}$ remains virtually the same (Table 3). The latter is somewhat unsurprising, as $E_{\mathrm{ES}}$ is mainly based on the hydrogen bonding network of the ammonium cation with the surrounding crown-ether oxygen atoms. Moreover, charge-transfer energies $E_{\mathrm{CT}}$, i.e., the mutual interactions between occupied and virtual spaces of the two components, become slightly more prevalent.

Table 4 displays the relative contributions of the electrostatic component $pE_{\mathrm{ES}}$ of the FMO-EDA calculations for all axle–counterion (**A1**-X) and axle–wheel (**A1**-**TTFC8**) interactions. For comparison, we provide the $\Delta G_{a}^{X}$ values of the anion attachment and the $\Delta G_{a}$ values of the **A1@TTFC8** formation reaction, respectively. To differentiate the two, an “X” was added in the denotation of the former.

**Figure 4:**
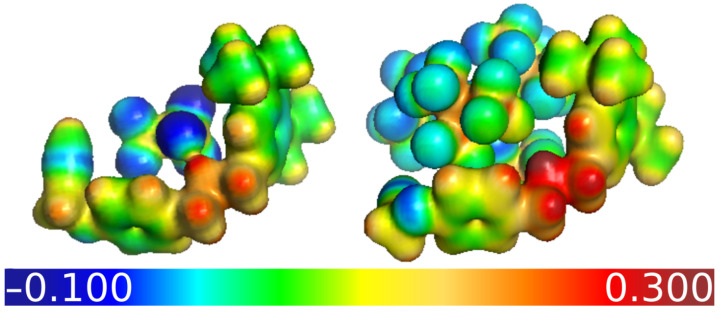
Electrostatic potential maps of **A1**-OTf (**left**) and **A1**-pf (**right**) in atomic units, with the isosurface value set to 0.02 a${}_{0}^{-3}$, obtained at the SMD/M06-2X/def2-TZVP level of theory. A color bar is added to indicate the potential at the surface.

In terms of the association of **A1** with the anions, the relative electrostatic contribution $pE_{\mathrm{ES}}$ to the binding is reduced from SCAs to WCAs. This is illustrated in Figure 4 using electrostatic potential maps. The strong electrostatic contribution in **A1**-OTf delocalises the charge from the ammonium centre to the anion, whereas the charge in **A1**-pf is more strongly localised. From SCAs to WCAs non-classical (quantum mechanical) contributions become more and more relevant. The FMO-EDA results suggest that the **A1**-X interactions can be mostly described by a classical electrostatic picture until the anions become too weakly coordinating, which is when their behaviour is much better described on a quantum–mechanical basis, with effects such as dispersion, charge-transfer, and exchange repulsion. It should be noted, however, that the electrostatic contribution is always the largest, regardless of the anion.

In contrast, $pE_{\mathrm{ES}}$ values for the **A1**-**TTFC8** binding hardly differ, if at all, upon variation of the anion. For no anion is the electrostatic contribution the largest one, but rather the dispersion and exchange portions dominate the binding situation (Table S9). In comparison to the **A1**-X interaction, the binding picture for **A1@TTFC8** can only be properly evaluated using quantum–mechanical terminology.

## 3. Materials and Methods

### 3.1. Syntheses

**Figure 5:**
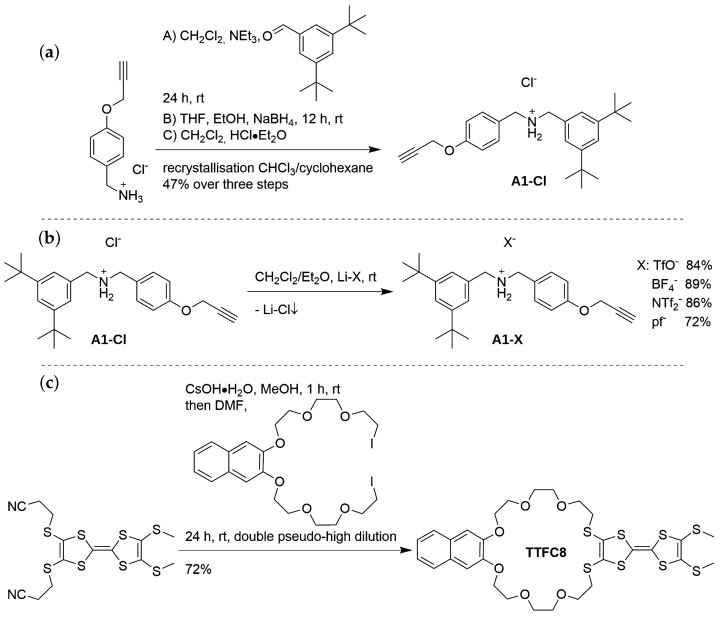
(**a**) Synthesis of **A1**-Cl by a modified procedure [63]. A: The imine formation between 3,5-di-tertbutyl-benzaldehyde and (4-(prop-2-yn-1-yloxy)phenyl)methanaminium chloride [64] is favoured by the binding of water to anhydrous sodium sulfate. B: Reduction of the formed imine by sodium tetrahydrido borate. C: **A1**-Cl is obtained by neutralisation of the amine with HCl·Et${}_{2}$O. The raw salt is purified by repeated recrystallisation from CHCl${}_{3}$/cyclohexane 1:4. (**b**) The counter anion exchange is realised through a double displacement reaction of **A1**-Cl with the respective lithium salt of the anion. Precipitation of lithium chloride shifts the equilibrium towards the highly soluble **A1**-X salts. (**c**) The reported synthesis of the macrocycle **TTFC8** [63] was adapted by adding both macrocycle precursors, at a very slow rate, to the reaction mixture.

The synthesis of **A1**-Cl has been previously reported [63]. The procedure was slightly modified, in order to improve the yield and obtain analytically pure **A1**-Cl on a gram scale (Figure 5a). Different counterions were introduced by metathesis reactions, using lithium salts of the required anion (Figure 5b). As all of these lithium salts are soluble in diethyl ether, in which lithium chloride is sparingly soluble, precipitation of the latter from the reaction mixture ensured completeness of the ion exchange. The obtained axle salts were purified of solvent traces by evaporation from tetrachloroethylene, and through subsequent removal of the latter in a fine vacuum. The reported synthesis of the macrocycle **TTFC8** [63] was modified by adding both macrocycle components under pseudo high-dilution conditions, thus increasing the yield to 72% (Figure 5c). More details regarding the synthetic procedures are found in the ESI (Sections S1 and S2).

### 3.2. ITC Experiments of MCAs and WCAs (BF${}_{4}^{-}$, NTf${}_{2}^{-}$, pf${}^{-}$)

**Figure 6:**
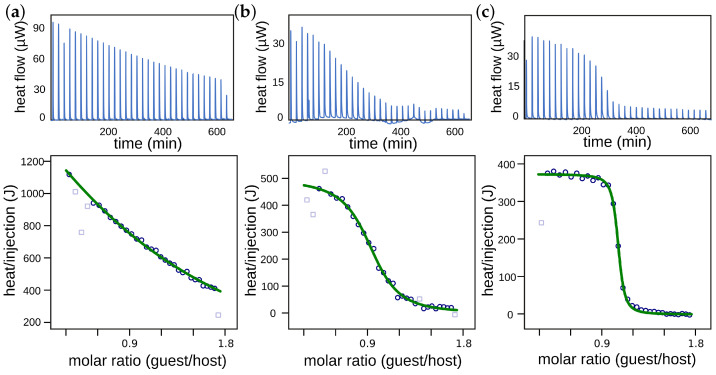
Titration plots (heat flow versus time and heat/volume versus guest/host ratio) obtained from ITC experiments at 298 K in 1,2-dichloroethane: (**a**) vial: **TTFC8**, syringe: **A1**-BF${}_{4}$; (**b**) vial: **TTFC8**, syringe: **A1**-NTf${}_{2}$; (**c**) vial: **TTFC8**, syringe: **A1**-pf. Points marked with non-filled squares were not considered in the fitting process.

ITC titrations (Figure 6) were carried out in dry 1,2-dichloroethane (DCE) at 298 K on a TAM III microcalorimeter (Waters GmbH, TA Instruments, Eschborn, Germany,). DCE is a suitable solvent, as pseudorotaxanes from crown ethers and ammonium axles are well bound in moderately polar solvents. It has the advantage, for example, over solvents such as DCM, in that it is non-volatile, which would otherwise cause significant errors in ITC experiments [44]. For **A1**-NTf${}_{2}$ and **A1**-pf, 800 μL of a 1.09 mM solution of **TTFC8** was placed in the sample cell, and 256 μL of an 8.00 mM solution of the ammonium salt was put into the syringe. For **A1**-BF${}_{4}$ 800 μL of a 10.9 mM solution of **TTFC8** was placed in the sample cell, and 256 μL of an 80.0 mM solution of the ammonium salt was put into the syringe. The titrations consisted of 32 consecutive injections of 8 μL, each with a 20 min interval between the injections. The heat of dilution was determined by the titration of ammonium salt solutions into the sample cell containing a blank solvent, and was subtracted from each data set. The heat flow generated in the sample cell was measured as a differential signal between the sample and reference cell. Hence, an exothermic event results in a positive heat flow and an endothermic event results in a negative heat flow. The data was analysed using the instrument’s internal software package, and fitted with a 1:1 binding model. Each titration was conducted at least three times, and the measured values for *K*, ΔH and ΔG were averaged. Binding data of **A1**-PF${}_{6}$ and **A1**-BArF${}_{24}$ are published elsewhere [44].

### 3.3. NMR Investigations of SCAs (BF${}_{4}^{-}$, OTf${}^{-}$)

The binding behaviour of **A1**-Cl and **A1**-OTf was studied by NMR spectroscopy, as the binding constants are out of the range in which they could be accurately determined by ITC. Lower binding affinities can be detected using NMR titration experiments. ${}^{1}$H-NMR spectra of samples containing 1 equiv. of macrocycle **TTFC8** (4.4 mM) and 63 equiv. of axle salt (277.3 mM), respectively, in 0.6 mL of CD${}_{2}$Cl${}_{2}$, were measured at 20 °C, 600 MHz. No significant signal shifts were observed, which is attributed to very weak or no binding between the two free components under these conditions. This observation is consistent with similar reported cases [39,40]. More details about the analytical procedure can be found in the ESI (Section S4).

### 3.4. Computational Details

Conformational sampling was conducted using the standalone CREST programme [65], based on the GFN2-xTB code [66] published by Grimme and co-workers. For a more adequate treatment of the conformers, the ALPB solvation model [67] with dichloromethane (DCM) as a solvent was employed. Note that DCE is currently not parametrised for this model. An initial CREST run was conducted for each combination of reactants (axles) and products (pseudorotaxanes, rotaxane), boith with every counterion and without. The results of the runs without counterions were used as a basis for the runs with counterions. Anions were placed close to the polarised hydrogen atoms and as close to the positively charged ammonium centre as possible. Initial test calculations, however, suggest that the starting position of the anion has little to no influence on the resulting set of conformations obtained through the CREST runs. This is furthermore supported by our observation that the most stable conformations of **A1@TTFC8** and **A1${}^{s}$@TTFC8** are retained irrespective of the counterion used. Macrocycles were evaluated without counterions. To save computational resources, loose parameters were applied by choosing an RMSD value for the resulting conformations of 2.0 Å, in addition to the “–quick” option of the programme. These parameters were benchmarked in accordance with the default parameters for the axle without a counterion and with Cl${}^{-}$ and OTs${}^{-}$. Results were deemed to be sufficiently similar.

Due to the more loose criteria, a more manageable subset of conformations (usually 50–100) was produced. The structures were then subjected to visual inspection, in order to obtain a smaller collection of the five or so conformers that seemed most relevant and distinguishable, which was used for further analyses. Figure S19 gives an example for the chosen conformers for **A1@TTFC8** in the presence of Cl${}^{-}$. Subsequent structure re-optimisations were performed at the PBEh-3c [68] level of DFT, which has been shown to yield very good results for these kinds of systems [69], in combination with the COSMO solvation model [70] using the Turbomole programme package (version 7.4) [71]. Normal modes to obtain $\Delta G_{\mathrm{therm}}$ were calculated using the single-point-Hessian (SPH) approach [57], together with the ALPB solvation model and using DCM as a solvent. Final single-point calculations to evaluate $\Delta E_{\mathrm{el}}$ were conducted at the PBE0-D3(BJ) [50,51,52], M06-2X [42], and ωB97X-D3 [53] levels of DFT, in combination with the SMD solvation model [41] and the def2-TZVP basis set [43] using the ORCA programme package (version 5.0.1) [72]. To stay consistent, DCM was used with the SMD solvent model, as DCE was not available for the SPH calculation. Differences between single-point calculations with DCM and DCE, used with SMD, amounted to roughly 2 kJ/mol in test calculations, which was deemed acceptable.

The mathematical differences of the employed functions amount to the treatment of the exchange-correlation kernel, with PBE0 being a classical GGA hybrid with 25% Hartree-Fock exchange, M06-2X being a so-called meta-GGA hybrid with 54% Hartree-Fock exchange, and ωB97X being a range-separated hybrid function with 100% Hartree-Fock exchange, in the long-range limit. Furthermore, the SPH calculations and the choice of the basis set were benchmarked to more high-level approaches, by comparing the former to normal modes obtained at the PBEh-3c level, and the latter to results from def2-QZVP calculations. Errors, overall, amounted to no more than 5–6 kJ/mol, and seemed to be of a systematic nature.

Results for the pseudorotaxane and (hypothetical) rotaxane formation without counterions and with Cl${}^{-}$ were compared to calculations, where $\Delta E_{\mathrm{el}}$ was obtained in vacuo, and we additionally computed $\Delta G_{\mathrm{solv}}$ instead, using the COSMO-RS approach [58] with its fine parametrisation utilising the Cosmotherm programme [73]. Resulting Gibbs free association energies when no counterions were present are quite off, which is not surprising, as COSMO-RS may yield unreasonable results if charged species are evaluated [54,59,60]. On the other hand, results for the Cl${}^{-}$ complexes agreed very well, with deviations below 3 kJ/mol.

Finally, to gain insight into the binding properties of the supramolecules and contact ion pairs **A1**-X, the FMO-EDA method [37,38], available in the GAMESS programme package [74], was utilised at the ωB97XD/cc-pVDZ [62] level. Note that a slightly different combination of method and basis set was employed, due to their availability in the GAMESS programme package and to save computational resources. The approach is based on an energy decomposition formula, and enables the evaluation of various contributions to intermolecular interactions.

## 4. Conclusions

In summary, we have investigated the structural, electronic, and thermodynamic properties of a pseudorotaxane, a rotaxane and their respective formation reactions in the presence of different anions, varying in their coordination ability. A major focus was put on Gibbs free association energies and the electronic interactions within **A1@TTFC8** and **A1${}^{s}$@TTFC8**. We have used ITC and ${}^{1}$H NMR measurements to validate our quantum–chemical approach which employed state-of-the-art theoretical calculations, based on GFN2-xTB and different density functional approximations. Additionally, we have utilised the FMO-EDA approach to analyse intermolecular interactions.

Upon investigating the conformational landscapes depending on the different anions, it was shown that a noticeable effect is exerted by the anions on the PES. However, due to the strong inter-component interactions of **A1@TTFC8** and **A1${}^{s}$@TTFC8** the most stable co-conformation, which is already present without any counterion, is retained in all other calculations. Calculated trends for $\Delta G_{a}$ are in general agreement with experiment and support our understanding of the anions’ coordination character. It was demonstrated that $\Delta G_{a}$ trends majorly depend on the stabilisation of the axle, which furthermore underlines the importance of including explicit counterions in quantum-chemical calculations.

Moreover, analysing the correlation between $\Delta G_{a}$ and the anions’ solvation free energies $\Delta G_{\mathrm{solv}}$ proved useful to extract physico-chemical parameters, such as the optimal association free energy $\Delta G_{a}^{\mathrm{opt}}$ and reaction constants $k_{1}$ and $k_{2}$, used as a measure of the influence of WCAs on the reaction. In order to extract meaningful information from these parameters, however, studying other reaction types is necessary. While beyond the scope of this work, it would certainly be interesting to address this in future studies.

Lastly, the calculation of structural penalties and application of the FMO-EDA method provided useful insight into the axle–wheel interaction in **A1@TTFC8** and **A1${}^{s}$@TTFC8**. Electrostatic interactions play a major role in almost all cases. However, when anions are more weakly coordinating, quantum–mechanical phenomena such as dispersion, charge transfer, and exchange repulsion become more relevant.

As applications of WCAs are manifold, so are the different perspectives on how to evaluate their features [6,7]. Studies such as the one at hand clearly show that there is potential for a more general description of the coordination properties of WCAs, or any anion for that matter, by utilising appropriate quantum–chemical methods.

## Figures and Tables

**Table 1:** Experimental and theoretical $\Delta G_{a}$ values with respect to the involved anion (X), obtained from ITC experiments and calculations at the SMD/M06-2X/def2-TZVP [41,42,43] level, respectively. All data are given in kJ/mol.

	SCAs			MCAs			WCAs	
X	Cl${}^{-}$	OTs${}^{-}$	OTf${}^{-}$	BF${}_{4}^{-}$	NTf${}_{2}^{-}$	PF${}_{6}^{-}$	BArF${}_{24}^{-}$	pf${}^{-}$
$\Delta G_{a,\exp.}$	≈0 ^1^	— ^2^	≈0 ^1^	−8.2	−24.2	−25.7 ^3^	−32.2 ^3^	−32.2
$\Delta G_{a,\mathrm{theo}.}$	−7.9	+0.8	−13.0	−25.5	−23.6	−29.3	−30.1	−33.3

^1^ not accurately determinable from experiment, ^2^ not measured, ^3^ from literature [44].

**Table 2:** Results obtained from the analysis of Equation (3) for **A1@TTFC8** and **A1${}^{s}$@TTFC8**, computed at the SMD/M06-2X/def2-TZVP level and from experimental values. $\Delta G_{a}^{\mathrm{opt}}$ values are given in kJ/mol. $k_{1}$ and $k_{2}$ are dimensionless. Note that BF${}_{4}^{-}$ had to be omitted from the analysis of **A1${}^{s}$@TTFC8**, as otherwise no convergence of the determination coefficients could be achieved.

	A1@TTFC8${}_{\exp.}$	A1@TTFC8${}_{\mathrm{theo}.}$	A1${}^{s}$@TTFC8
$\Delta G_{a}^{\mathrm{opt}}$	−33.3	−33.8	−38.7
$k_{1}$	0.042	0.067	0.149
$k_{2}$	0.058	0.051	0.043

**Table 3:** Change in energetic contributions to the inter-component interaction in **A1@TTFC8** and **A1${}^{s}$@TTFC8**. All data are given in kJ/mol.

	$E_{\mathrm{ES}}$	$E_{X}$	$E_{\mathrm{CT}}$	$E_{\mathrm{Disp}}$
**A1@TTFC8**	−357.4	375.8	−142.3	−390.8
**A1${}^{s}$@TTFC8**	−356.0	451.9	−162.7	−481.2

**Table 4:** FMO-EDA results obtained at the ωB97XD/cc-pVDZ [62] level, in comparison to Gibbs association free energies obtained at the SMD/M06-2X/def2-TZVP level, in terms of the interaction of **A1** with an anion X ($\Delta G_{a}^{X}$), and the inter-component interaction between **TTFC8** and **A1** in **A1@TTFC8** ($\Delta G_{a}$), in the presence of an anion (each given in kJ/mol). $pE_{\mathrm{ES}}$ is the ratio between the electrostatic contribution and all other stabilising effects in the FMO-EDA scheme, $pE_{\mathrm{ES}}=E_{\mathrm{ES}}/\left(E_{\mathrm{ES}}+E_{\mathrm{CT}}+E_{\mathrm{Disp}}\right)$.

X	Cl${}^{-}$	OTs${}^{-}$	OTf${}^{-}$	BF${}_{4}^{-}$	NTf${}_{2}^{-}$	PF${}_{6}^{-}$	BArF${}_{24}^{-}$	pf${}^{-}$
$\Delta G_{a}^{X}$(**A1**-X)	−56.8	−30.0	−14.5	−11.0	4.0	−5.2	19.9	26.8
$pE_{\mathrm{ES}}$(**A1**-X)	0.82	0.70	0.73	0.80	0.69	0.75	0.47	0.55
$\Delta G_{a}$(**A1@TTFC8**)	−7.9	0.8	−13.0	−25.5	−23.6	−29.3	−30.1	−33.3
$pE_{\mathrm{ES}}$(**A1@TTFC8**)	0.37	0.38	0.38	0.39	0.39	0.38	0.40	0.40

## Data Availability

See Supplementary Materials. Further data can be requested from the authors.

## Supplementary Materials

The following supporting information can be downloaded at: https://www.mdpi.com/article/10.3390/molecules28073077/s1, Section S1: General methods; Section S2: Synthetic Procedures; Section S3: NMR-Spectra; Section S4: NMR-investigation of SCAs (Cl${}^{-}$, OTf${}^{-}$); Section S5: Additional Computational results; Section S6: Computational results of **A2@BC7**.
