# Supplementary material for: The Interplay of Weakly Coordinating Anions and the Mechanical Bond: A Systematic Study of the Explicit Influence of Counterions on the Properties of (Pseudo)rotaxanes
Source: Molecules. 2023 Mar 30;28(7):3077. doi: 10.3390/molecules28073077 (PMC10096450; doi:10.3390/molecules28073077)
Supplement: Supplementary file 1 [file molecules-28-03077-s001.zip › ESI.pdf]

The Interplay of Weakly Coordinating Anions and the Mechanical Bond: A Systematic Study of the Explicit Influence of Counterions on the Properties of (Pseudo)rotaxanes

J. Felix Witte[1], Janos Wasternack[2], S. Wei[1], Christoph A. Schalley[2] and Beate Paulus[1],*
1 Institut für Chemie und Biochemie, Freie Universität Berlin, Arnimallee 22, 14195 Berlin, Germany
2 Institut für Chemie und Biochemie, Freie Universität Berlin, Arnimallee 20, 14195 Berlin, Germany
* Correspondence: jf.witte@fu-berlin.de (J.F.W.); b.paulus@fu-berlin.de (B.P.)

Table of Contents

Section S1: General methods **1**

Section S2: Synthetic Procedures **1**

Section S3: NMR-Spectra **9**

Section S4: NMR-investigation of SCAs (Cl^-, OTf^-) **19**

Section S5: Additional Computational results **20**

Section S6: Theoretical Calculations of **A2@BC7** **23**

References **25**

Section S1: General methods

NMR experiments were performed on JEOL ECX 400, JEOL ECP 500, Bruker AVANCE 500, JEOL ECZ 600 or Bruker AVANCE 700 instruments. Residual solvent signals were used as the internal standards. All shifts are reported in ppm and NMR multiplicities are abbreviated as s (singlet), d (doublet), t (triplet), m (multiplet) and br (broad).

High-resolution ESI mass spectra were recorded on an Agilent 6210 ESI-TOF mass spectrometer. HPLC grade solvents were used for sample preparation and the samples introduced into the ion source with a flow rate of 2-4 µL/min.

Section S2: Synthetic Procedures

All reagents and solvents were obtained from commercial sources and used without further purification. 3,5-di-tert-butylbenzaldehyde was purchased from abcr and used without further purification. Lithium tetrafluoroborate was purchased from Thermo Scientific, lithium triflate and lithium triflimidate were purchased from Sigma-Aldrich and each used without further purification. Lithium tetrakis(perfluoro-^{tert}butoxy)aluminate was provided by Prof. Ingo Krossing and Malte Sellin, University of Freiburg/Germany. Dry solvents were purchased from Acros Organics. Deuterated solvents were purchased from Eurisotop.

2,3-bis(2-cyanoethylthio)-6,7-bis(methylthio)tetrathiafulvalene[1,9], 2,3-bis(2-(2-(2-iodoethoxy)ethoxy)ethoxy)naphthalene[2], (4-(prop-2-yn-1-yloxy)phenyl)methanaminium chloride[3], were synthesized according to literature procedures. Thin-layer chromatography was performed on silica gel coated plates with fluorescent indicator F254 (Macherey-Nagel). For column chromatography, silica gel (0.04-0.063 mm, Macherey-Nagel) was used.

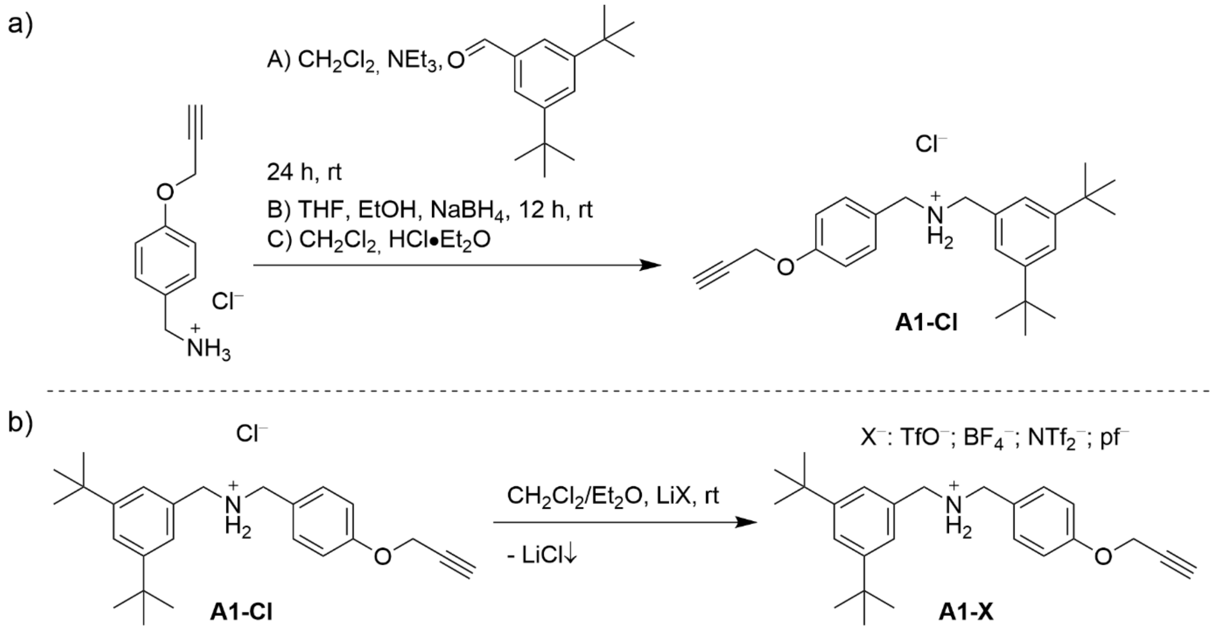

*Figure S 1: a) Modified synthesis of axle chloride **A1-Cl**.[4-6] b) Ion-metathesis between lithium salts and axle chloride, forming **A1-OTf**, **A1-BF$_4$**, **A1-NTf$_2$** or **A1-pf**. Quantitative ion exchange is driven by precipitation of lithium chloride.*

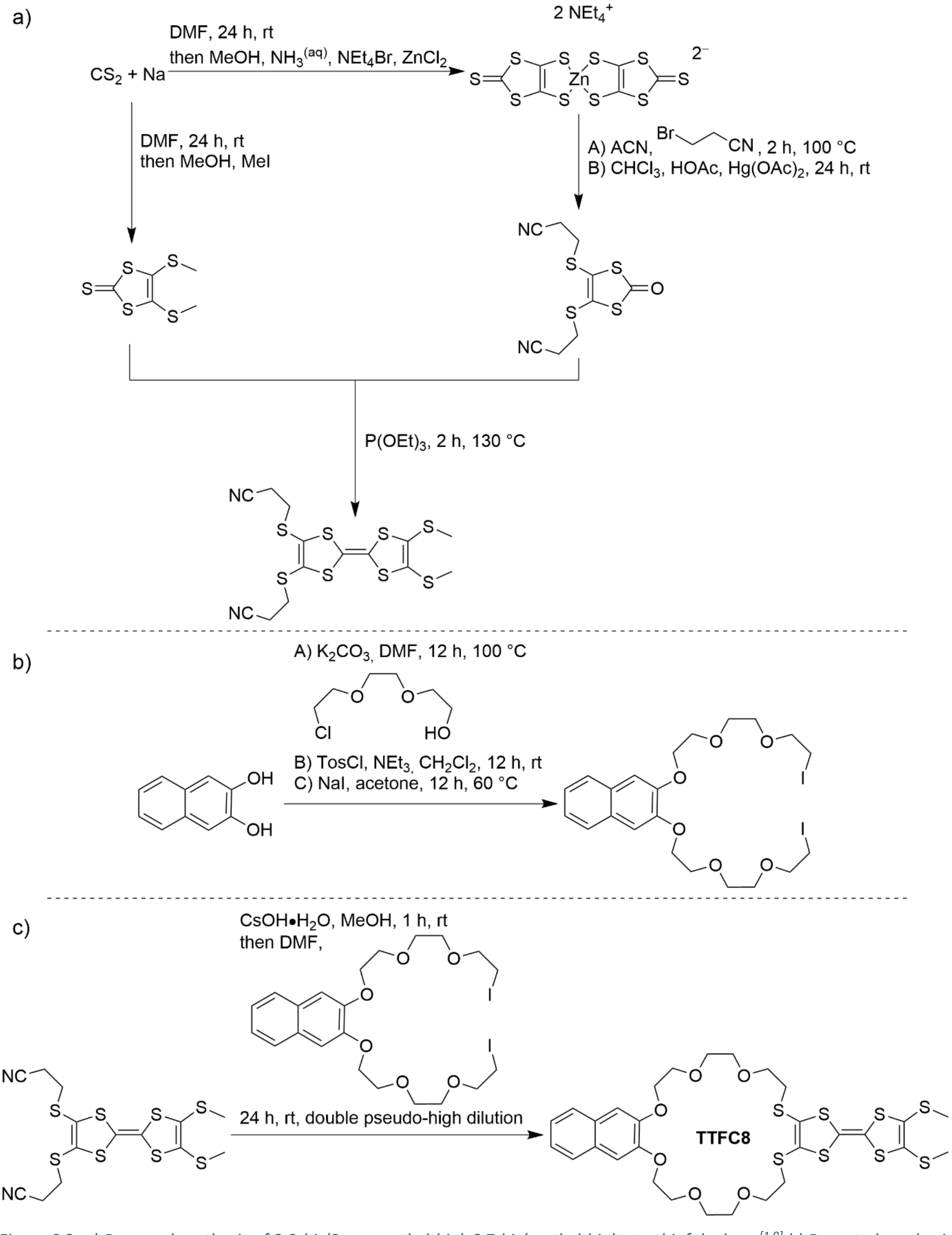

*Figure S 2: a) Reported synthesis of 2,3-bis(2-cyanoethylthio)-6,7-bis(methylthio)tetrathiafulvalene.[1,9] b) Reported synthesis of 2,3-bis(2-(2-(2-iodoethoxy)ethoxy)ethoxy)naphthalene.[2] c) Modified synthesis of macrocycle **TTFC8**.[4]*

2-(4,5-bis(methylthio)-1,3-dithiol-2-ylidene)-5,6,8,9,11,12,21,22,24,25,27,28-dodecahydro-[1,3]dithiolo[4,5-t]naphtho[2,3-h][1,4,7,10,13,16]hexaoxa[19,22]dithiacyclotetracosine (**TTFC8**)

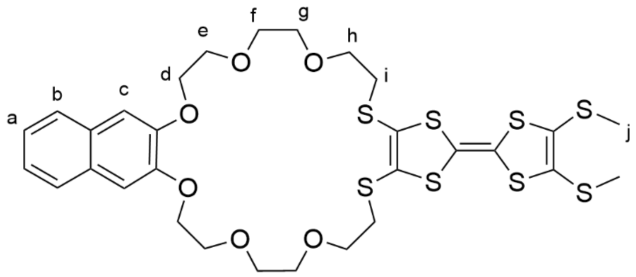

TTFC8

a) According to a modified literature procedure[4], 2,3-bis(2-cyanoethylthio)-6,7-bis(methylthio)tetrathiafulvalene (514 mg, 1.1 mmol, 1.1 equiv.) was dissolved in dimethylformamide (10 mL) and the solution was degassed by applying rough vacuum while the mixture was sonicated for 5 min. Caesium hydroxide monohydrate (406 mg, 2.3 mmol, 2.3 equiv.) was dissolved in methanol (10 mL) and the solution was degassed in the same manner as above. The clear, colourless caesium hydroxide solution was added in one portion to the orange solution of the cyanoethyl protected TTF-derivative and the solution was stirred for 1 h at room temperature. During that time, the colour of the mixture gradually changed from orange to dark red (solution A).

b) A solution of 2,3-bis(2-(2-(2-iodoethoxy)ethoxy)ethoxy)naphthalene (95% pure, 678 mg, 1.0 mmol, 1.0 equiv.) was prepared in dimethylformamide (20 mL) and subsequently degassed (solution B).

c) In a 250 mL SCHLENK round bottom flask, equipped with a stir bar and septum, dimethylformamide (20 mL) was degassed. Both solutions A & B are transferred into 24 mL syringes under a blanket of nitrogen. Both solutions were slowly added with syringe pumps in the course of 6 h (rate~3.6 mL/h) at room temperature and the resulting yellow-orange solution was further stirred at room temperature for 1 d.

The solvents were removed under reduced pressure. The residue was dissolved in dichloromethane (50 mL) and water (2 mL) was added and the resulting emulsion was vigorously stirred for 10 min. Anhydrous sodium sulphate (5 g) was added and the suspension was vigorously stirred for 10 min. The suspension was directly loaded onto a silica gel column packed with 100% dichloromethane. The first orange bands which eluted with dichloromethane were discarded. Subsequently the orange main band was eluted with 1% tetrahydrofuran in dichloromethane. The solvents were removed under reduced pressure, the resulting solid was dissolved in a minimal amount of dichloromethane (approximately 2 mL) and tetrachloroethylene (5 mL) was added. Both solvents were removed under reduced pressure and the solid residue was further dried in a fine vacuum for 1 d.

Analytically pure macrocycle **TTFC8** was obtained as salmon coloured solid (542 mg, 0.7 mmol, 72% yield), the analytical data is consistent with the reported values[4].

^{1}H NMR (600 MHz, CD_2Cl_2, ppm): δ = 7.67 (AA'BB' spin system, $^3J_{AX}$ = 6.1 Hz, 2H, a), 7.31 (AA'BB' spin system, $^3J_{AX}$ = 6.1 Hz, 2H, b), 7.13 (s: 2H, c), 4.23 (m: 4H, O-CH_2-CH_2-O, d), 3.93 (m: 4H, O-CH_2-CH_2-O, e), 3.78 (m: 4H, O-CH_2-CH_2-O, f), 3.68 (t, ^4J = 6.4 Hz, 4H, O-CH_2-CH_2-S, h), 3.66 (m: 4H, O-CH_2-CH_2-O, g), 3.00 (t, ^4J = 6.4 Hz, 4H, S-CH_2-CH_2-O, i), 2.41 (s: 6H, S-CH_3, j).

$^{13}C\{^{1}H\}$ NMR (125 MHz, CD_2Cl_2, ppm): δ = 149.4, 129.7, 128.7, 127.8, 126.6, 124.5, 111.5, 110.9, 108.3, 71.3, 71.0, 70.2, 70.0, 69.2, 36.1, 19.4.
HRMS (ESI): m/z calculated for $C_{30}H_{36}O_6S_8$ $[M+Na]^+$ 771.0175, found 771.0167.

N-(3,5-Di-tert-butylbenzyl)-1-(4-(prop-2-yn-1-yloxy)phenyl)methanaminium chloride (**A1-Cl**)

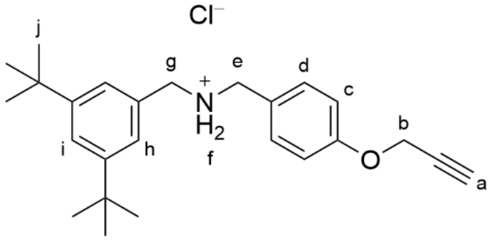

A1-Cl

a) According to modified literature procedures[4-6], a suspension of (4-(prop-2-yn-1-yloxy)phenyl)methanaminium chloride (9.4 g, 47 mmol, 1.00 equiv.), 3,5-di-*tert*-butylbenzaldehyde (11.0 g, 50 mmol, 1.05 equiv.), triethylamine (7.0 mL, 47 mmol, 1.00 equiv.) and anhydrous sodium sulphate (70.0 g, 493 mmol, 10.40 equiv.) in dichloromethane (250 mL) was stirred at room temperature for 2 d. The formation of the intermediate imine was confirmed by TLC (dichloromethane-cyclohexane 4:1; R_f^{imine}= 0.55). The mixture was filtered, and the precipitate washed with dichloromethane, the filtrates were combined, and the solvent was removed under reduced pressure.
b) The oily residue was dissolved in a mixture of anhydrous tetrahydrofuran (150 mL) and anhydrous ethanol (50 mL). Sodium tetrahydridoborate (3.6 g, 95 mmol, 2.01 equiv.) was added in portions at room temperature. The mixture was stirred at room temperature for 12 h. The progress of the reaction was monitored by TLC, indicating the absence of imine intermediate (dichloromethane-cyclohexane 4:1; R_f^{amine}= 0.00), and the presence of amine intermediate (dichloromethane-tetrahydrofuran 97.5:2.5; R_f^{amine}= 0.10). The solvents were removed under reduced pressure. The residue was suspended in dichloromethane, filtered and the precipitate washed with dichloromethane. The filtrates were combined, and the solvent was removed under reduced pressure. Raw amine intermediate was obtained as light-yellow oil (18.7 g).
c) The raw amine was dissolved in dichloromethane (200 mL) and $HCl\cdot Et_2O$ in diethyl ether (2 mol/L, 25 mL, 50 mmol, 1.06 equiv.) was added dropwise at room temperature. The acidity of the resulting clear solution was tested with wetted universal indicator paper (pH = 2). The solvents were removed under reduced pressure. The viscous residue was dissolved in a mixture of trichloromethane and cyclohexane (1:4, 200 mL) at 80 °C. The slightly turbid, hot mixture was immediately filtered through a fine porosity fritted funnel (Ø=6 cm). The filtrate was cooled to 4 °C for 1 d. The mixture was filtered, and the colourless precipitate was dried in a flow of air for 15 min. The cycle of dissolution in the hot solvent mixture, hot filtration, crystallisation at 4 °C and filtration was repeated five times.
Additional crops of product can be obtained by evaporation of the mother liquors and repeated crystallisation. Analytically pure axle chloride **A1-Cl** was obtained as colourless crystalline solid (11.41 g, 28.5 mmol, 60% yield over three steps).
^{1}H NMR (600 MHz, $CDCl_3$, ppm): δ = 10.31 (br: 2H, NH_2^+, f), 7.43 (AA'XX' spin system, $^3J_{AX}$ = 8.6 Hz, 2H, d), 7.40 (AA'X spin system, $^3J_{AX}$ = 1.5 Hz, 1H, i), 7.33 (AA'X spin system, $^3J_{AX}$ = 1.5 Hz,

2H, h), 6.93 (AA'XX' spin system, $^{3}J_{AX}$ = 8.6 Hz, 2H, c), 4.55 (d, ^{5}J = 2.4 Hz, 2H, CH_2, b), 3.87 (br: 2H, CH_2N, e), 3.77 (br: 2H, CH_2N, g), 2.46 (t, ^{5}J = 2.4 Hz, 1H, CH_{alkyne}, a), 1.33 (s: 18H, CH_3, j).
^{13}C{^{1}H} NMR (125 MHz, CDCl$_3$, ppm): δ = 158.2, 151.9, 132.1, 129.2, 124.7 123.2, 115.4, 78.3, 75.9, 55.8, 48.9, 47.7, 35.1, 31.6, 27.1.
HRMS (ESI): m/z calculated for $C_{25}H_{134}NO$ $[M]^+$ 364.2640, found 364.2650.

N-(3,5-Di-tert-butylbenzyl)-1-(4-(prop-2-yn-1-yloxy)phenyl)methanaminium trifluoromethyl sulfonate (**A1-OTf**)

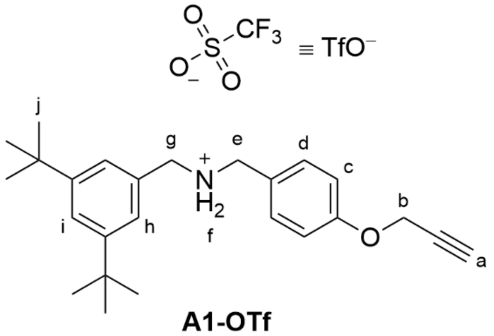

A1-OTf

Axle chloride **A1-Cl** (400 mg, 1.0 mmol, 1.0 equiv.) was dissolved in diethyl ether (10 mL) and lithium triflate (172 mg, 1.1 mmol, 1.1 equiv.) was dissolved in diethyl ether (10 mL). Both solutions were mixed at room temperature and stirred for 1 h. A very fine, colourless precipitate formed slowly. Celite (500 mg) was added, and the suspension was stirred for 20 min. The solvents were removed under reduced pressure and the solid was resuspended in dichloromethane (20 mL) and stirred for 20 min. The mixture was filtered over a plug of Celite (l=1.5 cm, Ø=2 cm), the solid was washed with dichloromethane and the solvents were removed under reduced pressure. The oily residue was dissolved in a minimal amount of dichloromethane and tetrachloroethylene (5 mL) was added, the solvents were removed under reduced pressure and the solid residue was dried in a fine vacuum for 1 d.
Analytically pure axle triflate **A1-OTf** was obtained as colourless solid (430 mg, 83.7 mmol, 84% yield).
^{1}H NMR (600 MHz, CDCl$_3$, ppm): δ = 9.36 (br: 2H, NH_2^+, f), 7.42 (br: 1H, i), 7.37c (AA'XX' spin system, $^{3}J_{AX}$ = 8.6 Hz, 2H, d), 7.27 (br: 2H, h), 6.94 (AA'XX' spin system, $^{3}J_{AX}$ = 8.6 Hz, 2H, c), 4.56 (d, ^{5}J = 2.4 Hz, 2H, CH_2, b), 3.92 (br: 2H, CH_2N, e), 3.83 (br: 2H, CH_2N, g), 2.47 (t, ^{5}J = 2.4 Hz, 1H, CH_{alkyne}, a), 1.32 (s: 18H, CH_3, j).
^{19}F NMR (565 MHz, CDCl$_3$, ppm): δ = −78.01 (s: 3F, CF_3).
^{13}C{^{1}H} NMR (125 MHz, CDCl$_3$, ppm): δ = 158.4, 152.1, 131.9, 129.1, 124.5, 123.5, 123.0, 115.5, 78.2, 76.0, 55.8, 49.6, 48.3, 35.1, 31.5.
HRMS (ESI): m/z calculated for $C_{25}H_{134}NO$ $[M]^+$ 364.2640, found 364.2622.

N-(3,5-Di-tert-butylbenzyl)-1-(4-(prop-2-yn-1-yloxy)phenyl)methanaminium tetrafluoroborate (**A1-BF$_4$**)

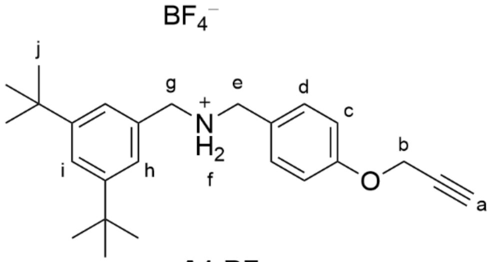

A1-BF$_4$

Axle chloride **A1-Cl** (400 mg, 1.00 mmol, 1.00 equiv.) was dissolved in dichloromethane (5 mL) and lithium tetrafluoridoborate (95 mg, 1.05 mmol, 1.05 equiv.) was dissolved in diethyl ether (10 mL). Both solutions were mixed at room temperature and stirred for 1 h. A very fine, colourless precipitate formed slowly. Celite (500 mg) was added, and the suspension was stirred for 20 min. The solvents were removed under reduced pressure and the solid was resuspended in dichloromethane (20 mL) and stirred for 20 min. The mixture was filtered over a plug of Celite (l=1.5 cm, Ø=2 cm), the solid was washed with dichloromethane and the solvents were removed under reduced pressure. The oily residue was dissolved in a minimal amount of dichloromethane and tetrachloroethylene (5 mL) was added, the solvents were removed under reduced pressure and the solid residue was dried in a fine vacuum for 1 d.
Analytically pure axle tetrafluoridoborate **A1-BF$_4$** was obtained as colourless solid (403 mg, 89.29 mmol, 89% yield).
^{1}H NMR (600 MHz, $CDCl_3$, ppm): δ = 9.41 (br: 2H, NH_2^+, f), 7.42 (br: 1H, i), 7.39 (AA'XX' spin system, $^{3}J_{AX}$ = 7.7 Hz, 2H, d), 7.30 (br: 2H, h), 6.94 (AA'XX' spin system, $^{3}J_{AX}$ = 7.7 Hz, 2H, c), 4.57 (d, ^{5}J = 2.4 Hz, 2H, CH_2, b), 3.94 (br: 2H, CH_2N, e), 3.85 (br: 2H, CH_2N, g), 2.47 (t, ^{5}J = 2.4 Hz, 1H, CH_{alkyne}, a), 1.33 (s: 18H, CH_3, j).
^{19}F NMR (565 MHz, $CDCl_3$, ppm): δ = −148.01 & −148.07 (4F, BF_4^-).
^{13}C{^{1}H} NMR (125 MHz, $CDCl_3$, ppm): δ = 158.4, 152.1, 132.0, 124.6, 123.4, 122.9, 121.9, 115.5, 78.2, 76.0, 55.8, 49.5, 48.2, 35.1, 31.5.
HRMS (ESI): m/z calculated for $C_{25}H_{134}NO$ $[M]^+$ 364.2640, found 364.2642; m/z calculated for BF_4 $[M]^-$ 87.0029, found 86.9972.

N-(3,5-Di-tert-butylbenzyl)-1-(4-(prop-2-yn-1-yloxy)phenyl)methanaminium bis((trifluoromethyl)sulfonyl)amide (**A1-NTf$_2$**)

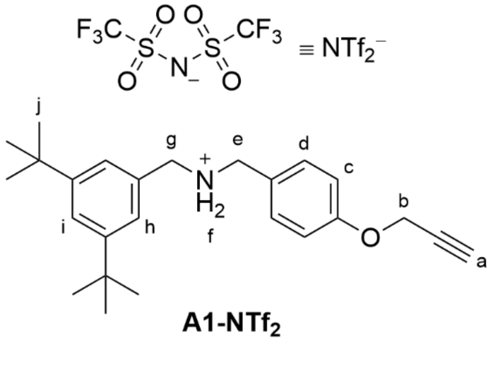

A1-NTf$_2$

Axle chloride **A1-Cl** (400 mg, 1.00 mmol, 1.00 equiv.) was dissolved in dichloromethane (5 mL) and lithium triflimidate (301 mg, 1.05 mmol, 1.05 equiv.) was dissolved in diethyl ether (10 mL). Both solutions were mixed at room temperature and stirred for 2 min. A very fine, colourless precipitate formed. Celite (500 mg) was added, and the suspension was stirred for 20 min. The solvents were removed under reduced pressure and the solid was resuspended in dichloromethane (20 mL) and stirred for 20 min. The mixture was filtered over a plug of Celite (l=1.5 cm, Ø=2 cm), the solid was washed with dichloromethane and the solvents were removed under reduced pressure. The oily residue was dissolved in a minimal amount of dichloromethane and tetrachloroethylene (5 mL) was added, the solvents were removed under reduced pressure and the solid residue was dried in a fine vacuum for 1 d.

Analytically pure axle triflimidate **A1-NTf$_2$** was obtained as colourless waxy solid (557 mg, 86.40 mmol, 86% yield).

^{1}H NMR (600 MHz, CDCl$_3$, ppm): δ = 7.51 (AA'X spin system, $^3J_{AX}$ = 1.8 Hz, 1H, i), 7.28 (AA'XX' spin system, $^3J_{AX}$ = 8.7 Hz, 2H, d), 7.15 (AA'X spin system, $^3J_{AX}$ = 1.8 Hz, 2H, h), 7.02 (AA'XX' spin system, $^3J_{AX}$ = 8.7 Hz, 2H, c), 4.70 (d, ^5J = 2.4 Hz, 2H, CH_2, b), 4.13 (br: 2H, CH_2N, e), 4.08 (br: 2H, CH_2N, g), 2.54 (t, ^5J = 2.4 Hz, 1H, CH_{alkyne}, a), 1.33 (s: 18H, CH_3, j).

^{19}F NMR (565 MHz, CDCl$_3$, ppm): δ = −78.60 (s: 6F, CF_3).

^{13}C{^{1}H} NMR (125 MHz, CDCl$_3$, ppm): δ = 159.0, 152.8, 131.5, 128.2, 124.4, 124.0, 121.9, 118.6, 116.0, 78.0, 76.2, 56.0, 50.7, 49.5, 35.1, 31.4.

HRMS (ESI): m/z calculated for $C_{25}H_{134}NO$ [M]$^+$ 364.2640, found 364.2612.

N-(3,5-Di-tert-butylbenzyl)-1-(4-(prop-2-yn-1-yloxy)phenyl)methanaminium tetrakis((1,1,1,3,3,3-hexafluoro-2-(trifluoromethyl)propan-2-yl)oxy)-l4-aluminate (**A1-pf**)

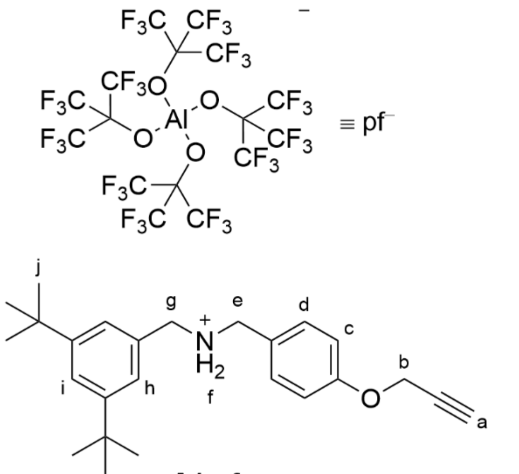

A1-pf

Axle chloride **A1-Cl** (400 mg, 1.00 mmol, 1.00 equiv.) was dissolved in dichloromethane (5 mL) and Li-pf (1020 mg, 1.05 mmol, 1.05 equiv.) was dissolved in diethyl ether (10 mL). Both solutions were mixed at room temperature and stirred for 2 min. A very fine, colourless precipitate formed immediately. Celite (500 mg) was added, and the suspension was stirred for 20 min. The solvents were removed under reduced pressure and the solid was resuspended in dichloromethane (20 mL) and stirred for 20 min. The mixture was filtered over a plug of Celite (l=1.5 cm, Ø=2 cm), the solid was washed with dichloromethane and the solvents were removed under reduced pressure. The oily residue was dissolved in a minimal

amount of dichloromethane and tetrachloroethylene (5 mL) was added, the solvents were removed under reduced pressure and the solid residue was dried in a fine vacuum for 1 d. Analytically pure axle perflouro-*tert*-butoxy-aluminate **A1-pf** was obtained as colourless waxy solid (960 mg, 72.18 mmol, 72% yield).

^{1}H NMR (600 MHz, $CDCl_3$, ppm): δ = 7.63 (AA'X spin system, $^{3}J_{AX}$ = 1.7 Hz, 1H, i), 7.27 (AA'XX' spin system, $^{3}J_{AX}$ = 8.7 Hz, 2H, d), 7.14 (AA'XX' spin system, $^{3}J_{AX}$ = 8.7 Hz, 2H, c), 7.11 (AA'X spin system, $^{3}J_{AX}$ = 1.7 Hz, 2H, h), 4.76 (d, ^{5}J = 2.4 Hz, 2H, CH_2, b), 4.32 (br: 4H, CH_2N, e & g), 2.55 (t, ^{5}J = 2.4 Hz, 1H, CH_{alkyne}, a), 1.33 (s: 18H, CH_3, j).

^{19}F NMR (565 MHz, $CDCl_3$, ppm): δ = −75.19 (s: 24F, CF_3).

^{13}C{^{1}H} NMR (125 MHz, $CDCl_3$, ppm): δ = 158.9, 153.9, 131.0, 127.4, 125.8, 123.4, 122.4, 120.4, 116.8, 76.5, 56.1, 53.3, 52.3, 35.2, 31.3.

HRMS (ESI): m/z calculated for $C_{25}H_{134}NO$ $[M]^+$ 364.2640, found 364.2613; m/z calculated for $C_{16}O_4AlF_{36}$ $[M]^-$ 966.9037, found 966.9190.

Section S3: NMR-Spectra

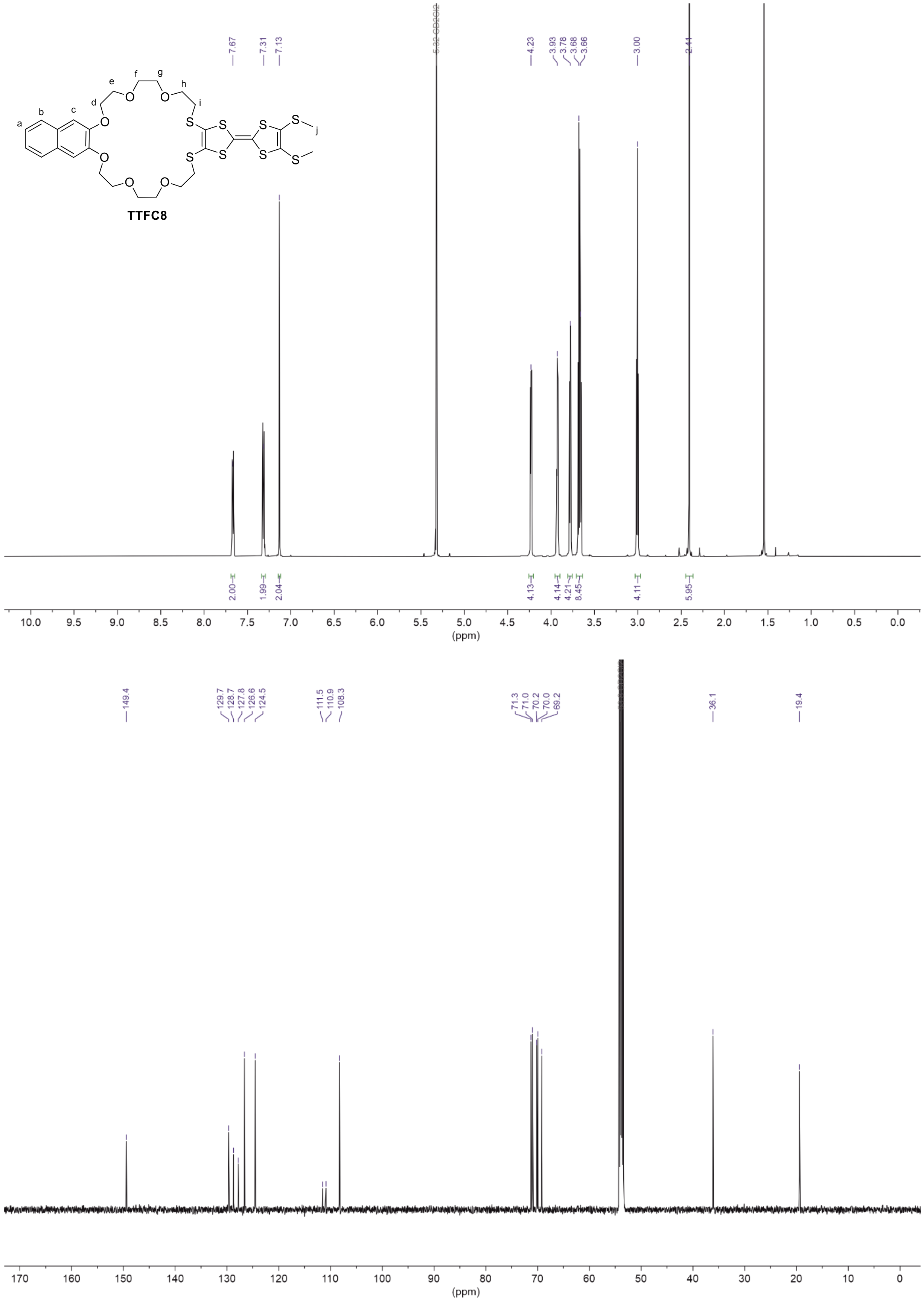

*Figure S 3 top: ^{1}H NMR-spectrum of **TTFC8**, 600 MHz, $CDCl_3$, 20°C. Bottom: $^{13}C\{^{1}H\}$ NMR-spectrum of **TTFC8**, 125 MHz, $CDCl_3$, 20°C.*

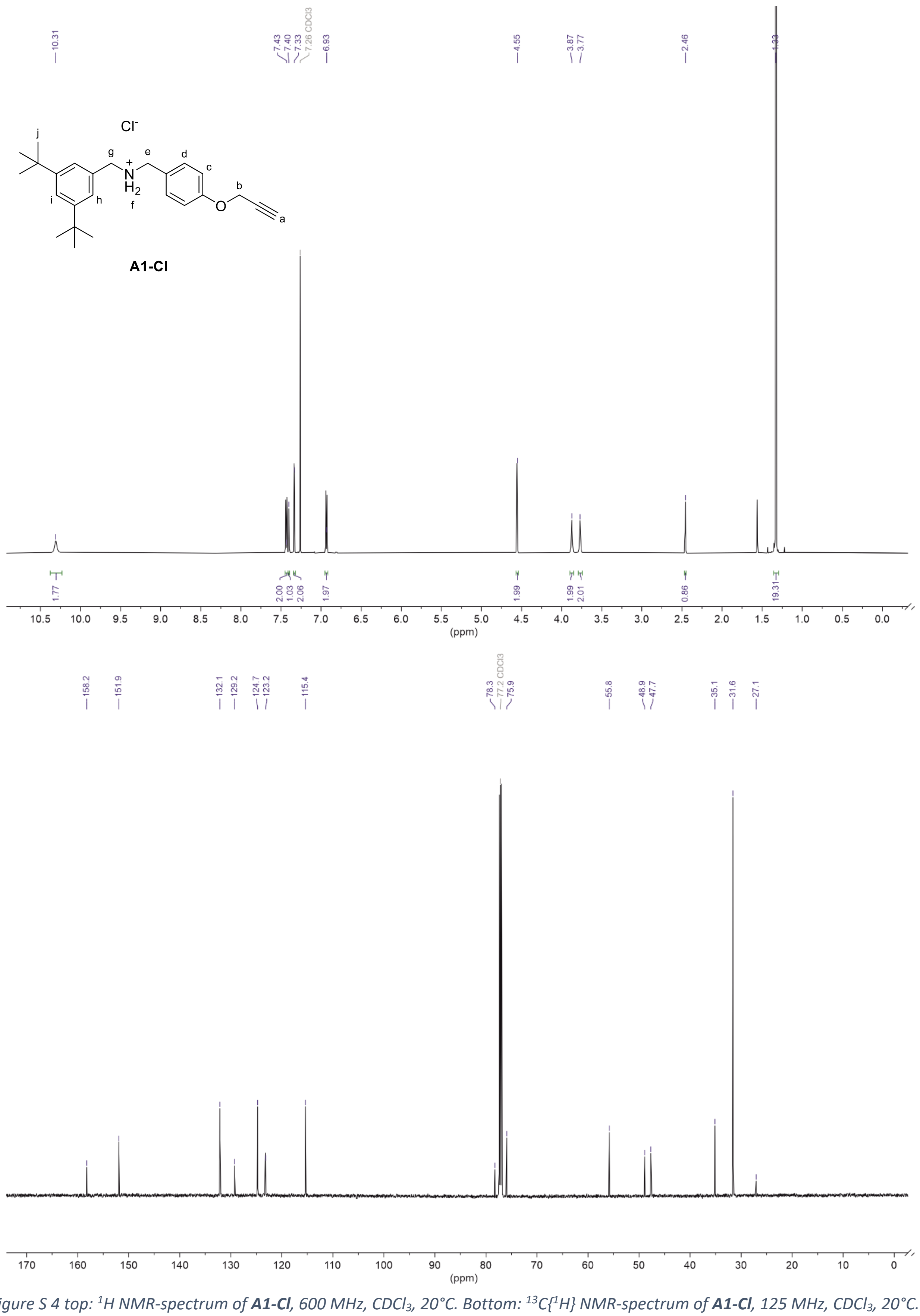

*Figure S 4 top: ^{1}H NMR-spectrum of **A1-Cl**, 600 MHz, $CDCl_3$, 20°C. Bottom: $^{13}C\{^{1}H\}$ NMR-spectrum of **A1-Cl**, 125 MHz, $CDCl_3$, 20°C.*

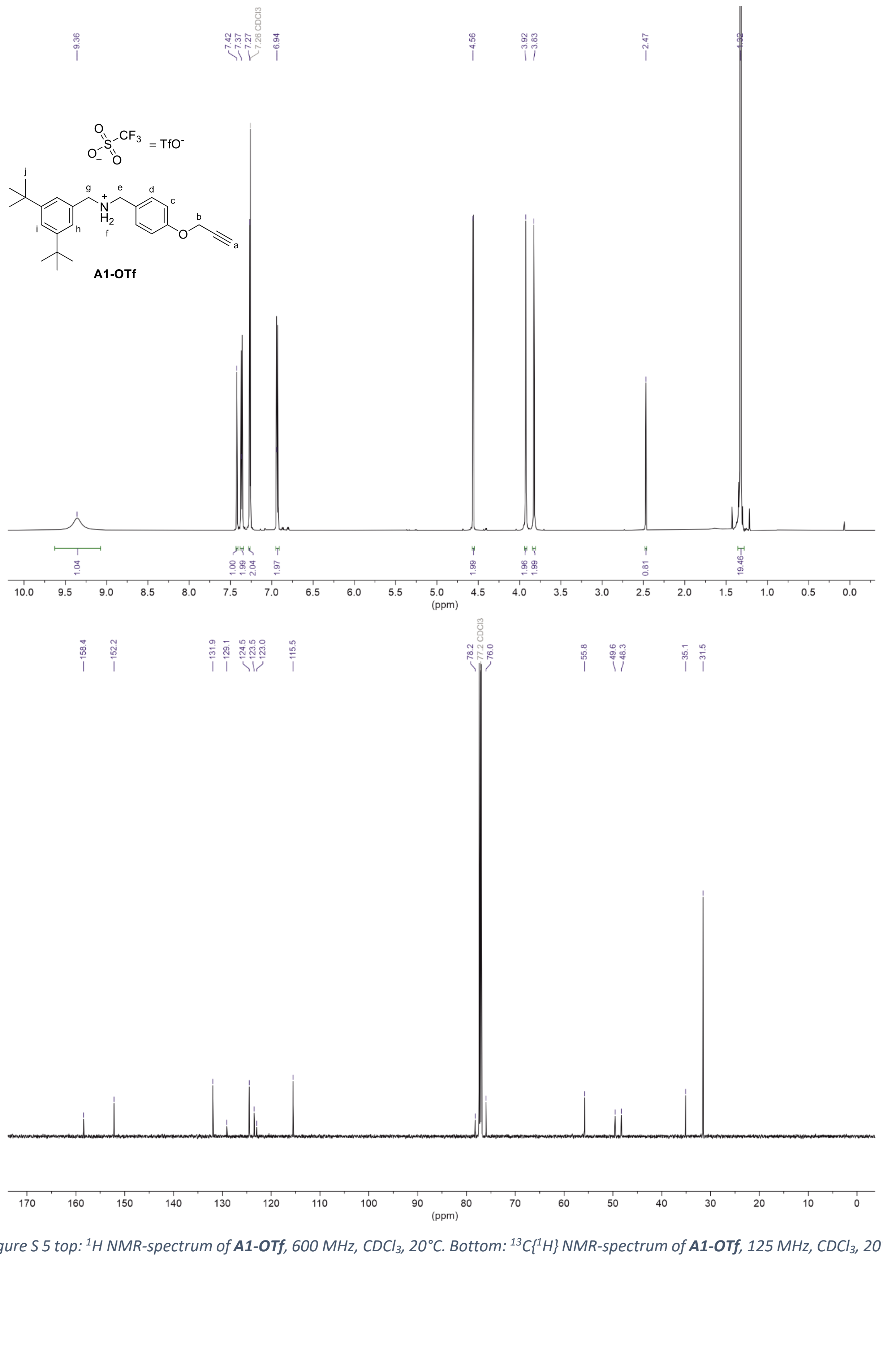

*Figure S 5 top: ^{1}H NMR-spectrum of **A1-OTf**, 600 MHz, $CDCl_3$, 20°C. Bottom: $^{13}C\{^{1}H\}$ NMR-spectrum of **A1-OTf**, 125 MHz, $CDCl_3$, 20°C.*

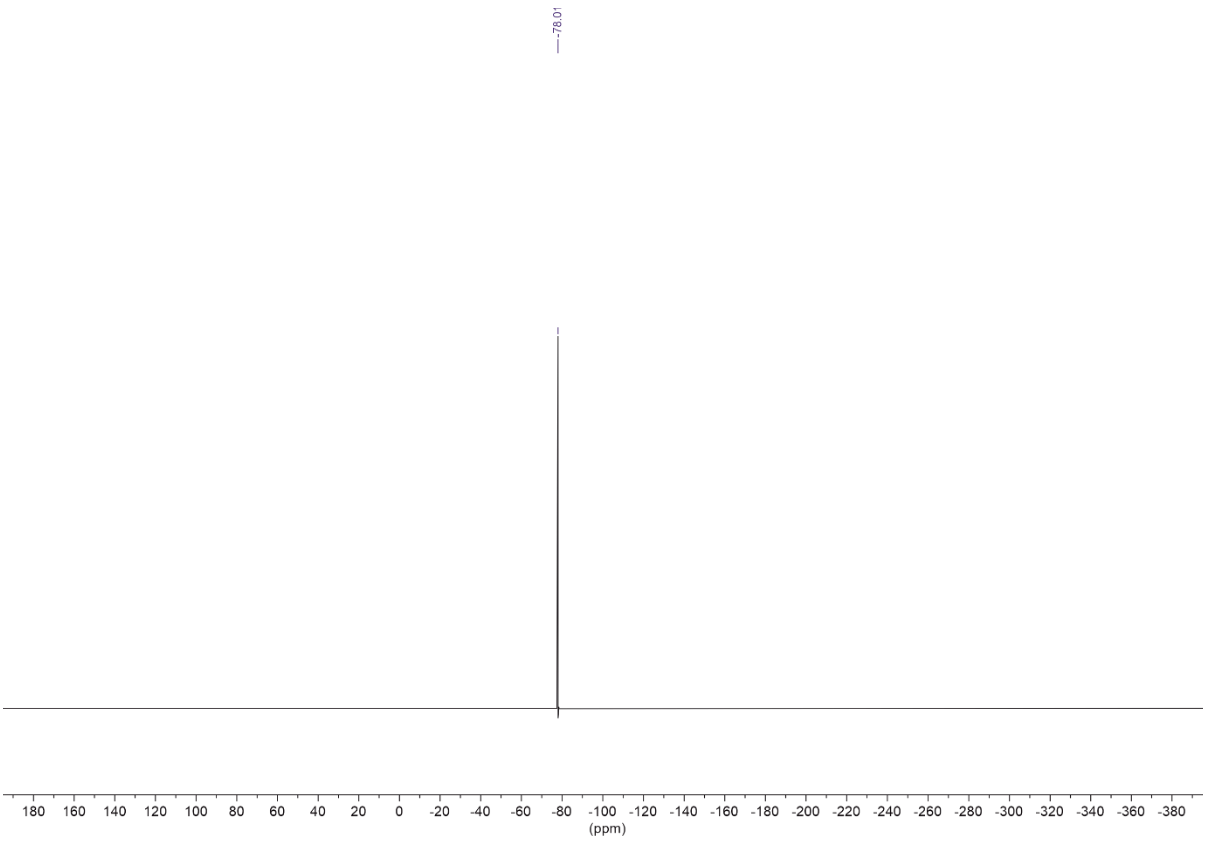

Figure S 6: ^{19}F NMR-spectrum of ***A1-OTf****, 471 MHz, $CDCl_3$, 20°C.*

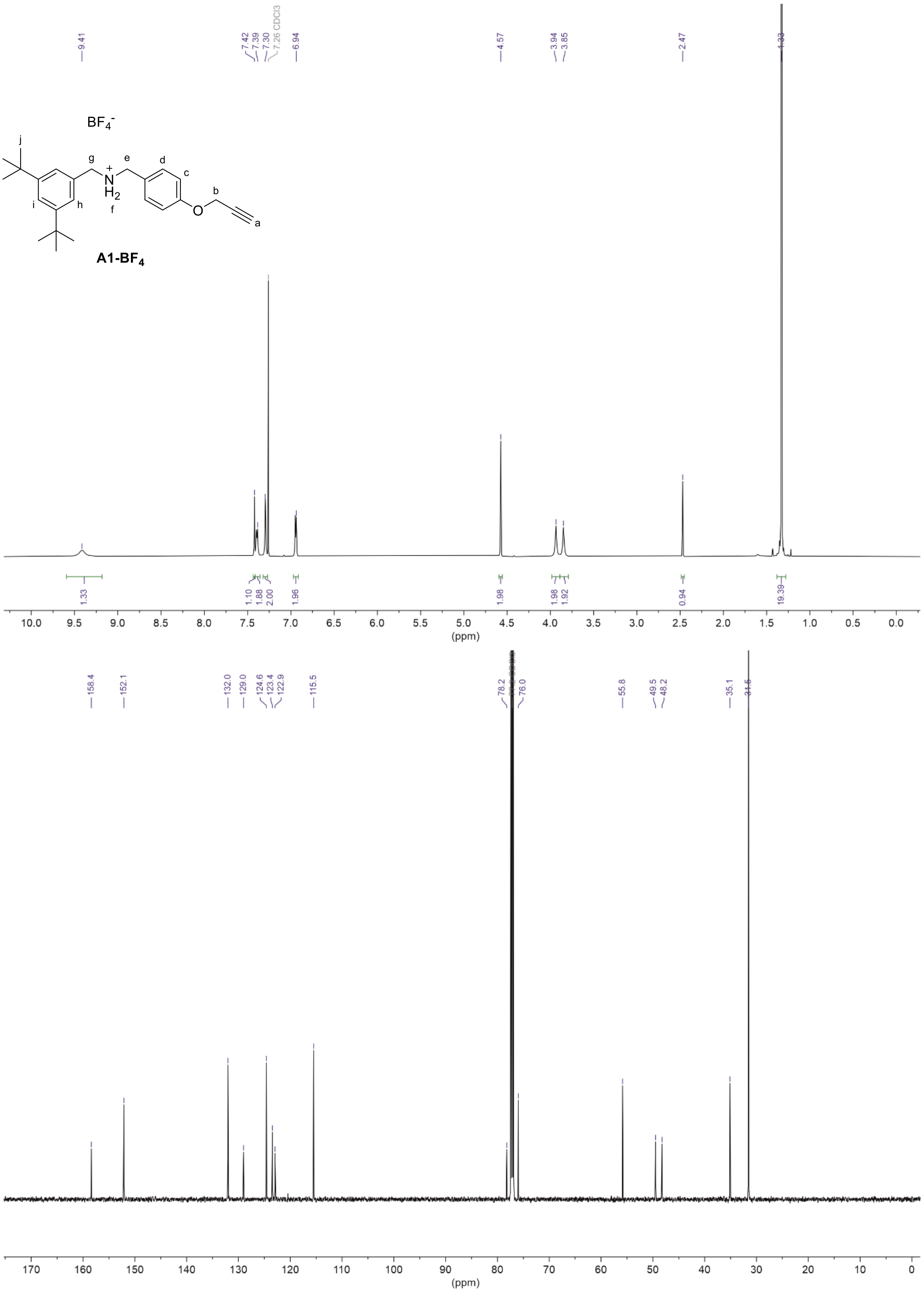

*Figure S 7 top: ^{1}H NMR-spectrum of **A1-BF$_4$**, 600 MHz, $CDCl_3$, 20°C. Bottom: $^{13}C\{^{1}H\}$ NMR-spectrum of **A1-BF$_4$**, 125 MHz, $CDCl_3$, 20°C.*

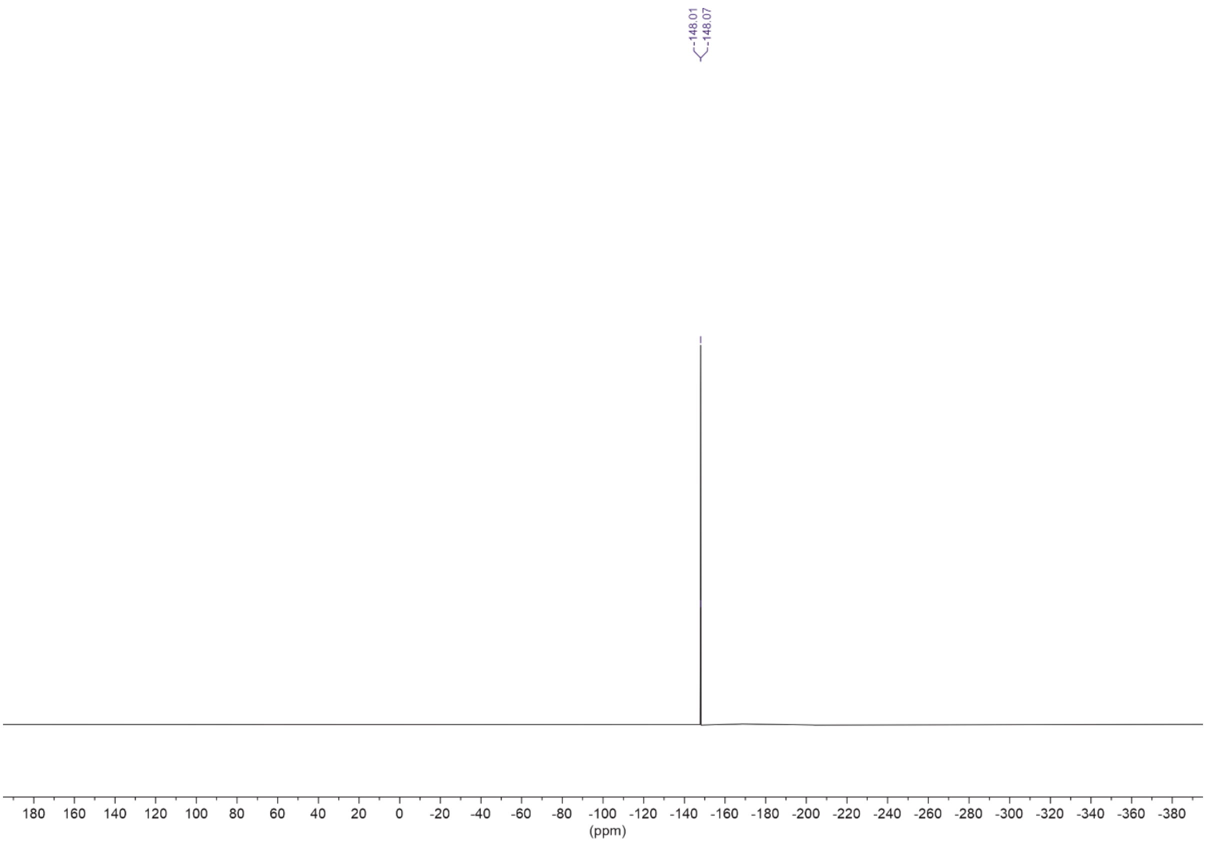

*Figure S 8: ^{19}F NMR-spectrum of **A1-BF$_4$**, 471 MHz, $CDCl_3$, 20°C.*

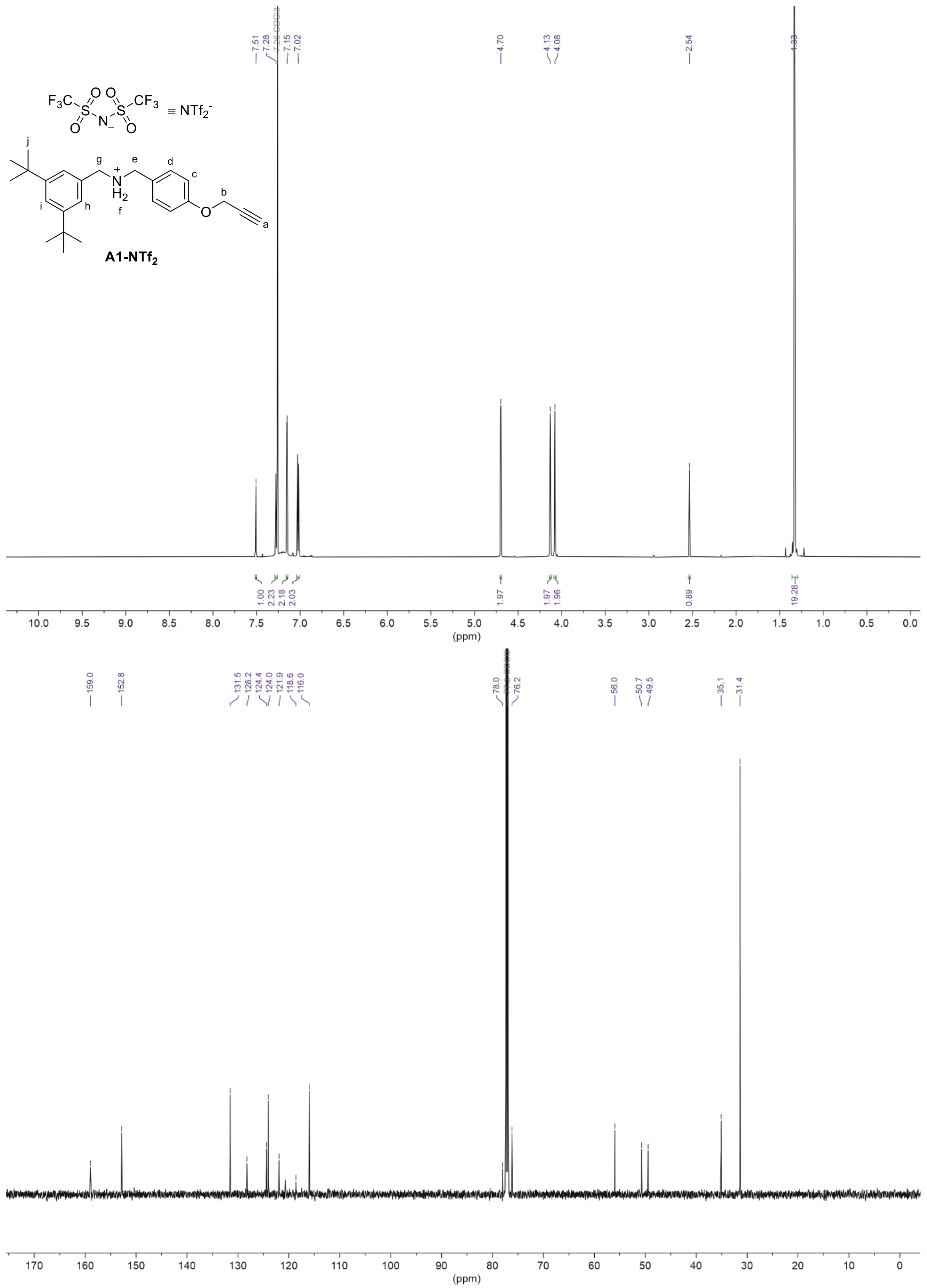

*Figure S 9 top: ^{1}H NMR-spectrum of **A1-NTf$_2$**, 600 MHz, CDCl$_3$, 20°C. Bottom: ^{13}C{^{1}H} NMR-spectrum of **A1-NTf$_2$**, 125 MHz, CDCl$_3$, 20°C.*

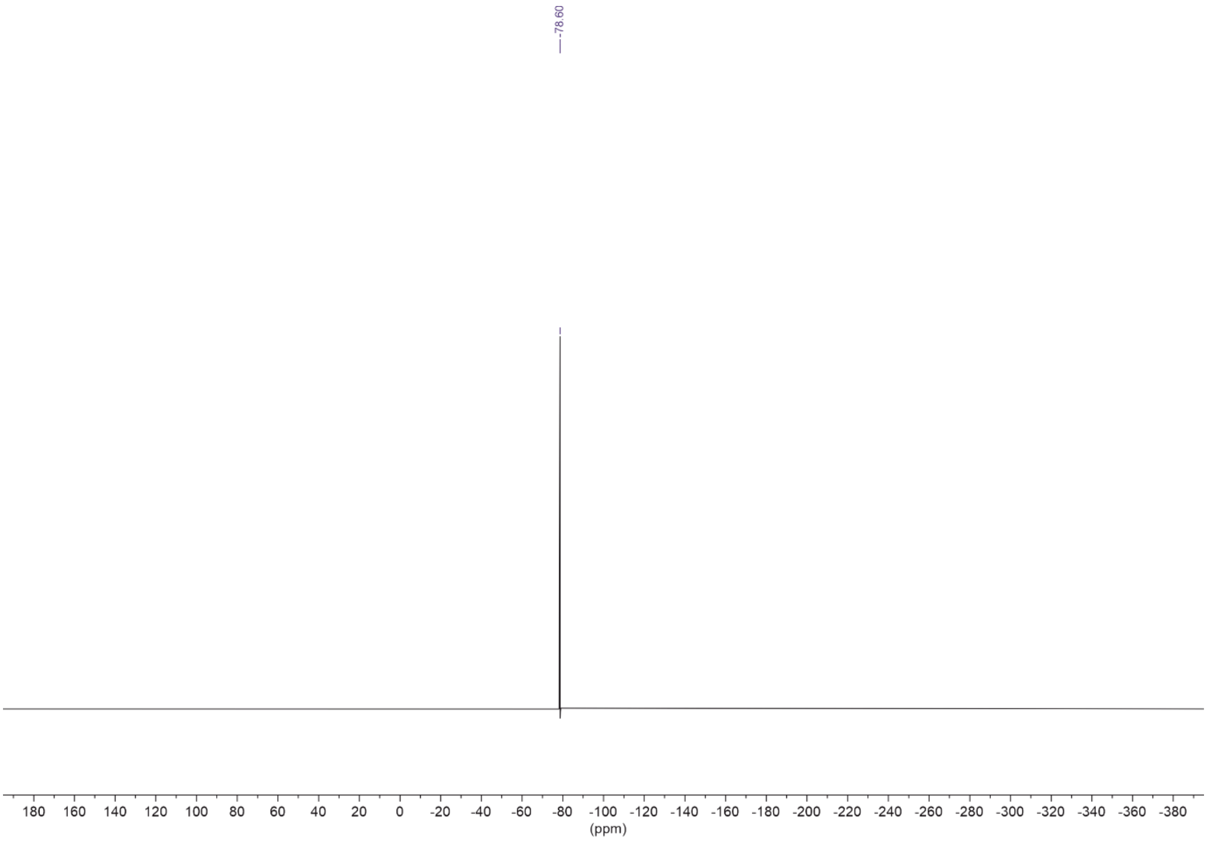

*Figure S 10: ^{19}F NMR-spectrum of **A1-NTf₂**, 471 MHz, $CDCl_3$, 20°C.*

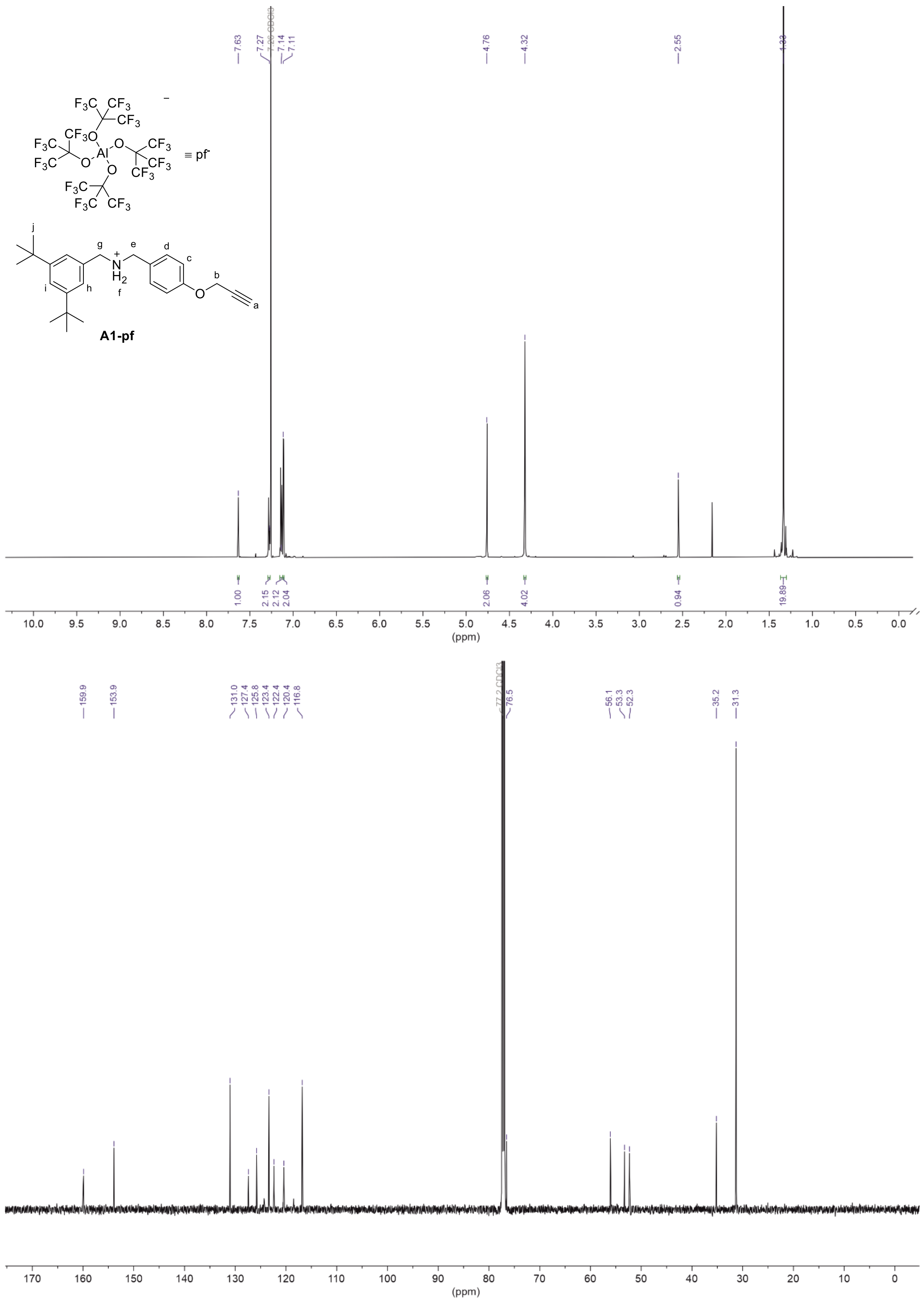

*Figure S 11 top: ^{1}H NMR-spectrum of **A1-pf**, 600 MHz, $CDCl_3$, 20°C. Bottom: $^{13}C\{^{1}H\}$ NMR-spectrum of **A1-pf**, 125 MHz, $CDCl_3$, 20°C.*

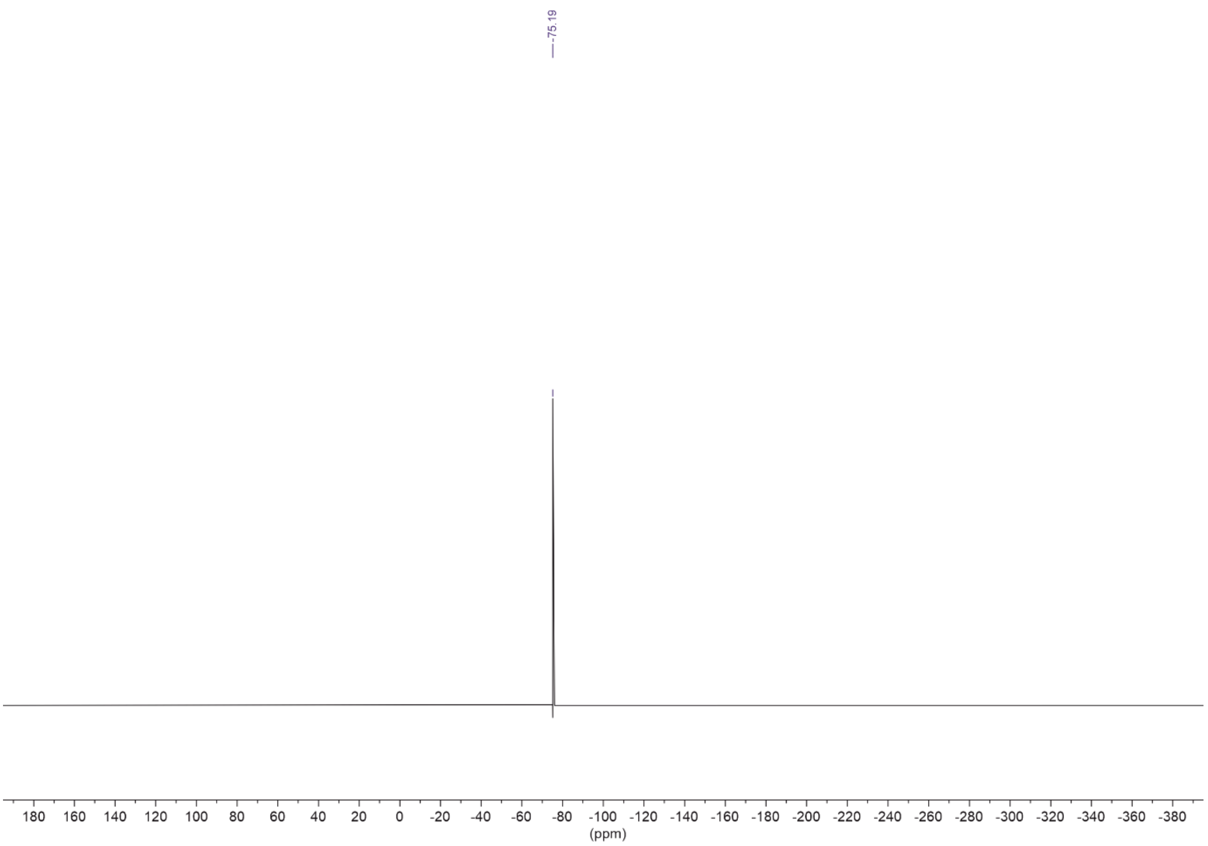

*Figure S 12: ^{19}F NMR-spectrum of **A1-pf**, 471 MHz, $CDCl_3$, 20°C.*

Section S4: NMR-investigation of SCAs (Cl^-, OTf^-)

The binding behaviour of **A1-Cl** and **A1-OTf** was studied by NMR. The binding constants of **A1** with these anions are out of the range in which they could be accurately determined by ITC. Therefore, NMR-experiments were conducted as lower binding affinities can be determined using NMR-titration experiments. As ITC provides the binding stoichiometry as well as the binding constant and enthalpy in one experiment, where possible, ITC is the preferred method to determine the thermodynamic properties of pseudorotaxane systems with moderate to high binding constants. ^{1}H NMR spectra of samples containing 1 equiv. of macrocycle (4.4 mM) and 63 equiv. of axle salt (277.3 mM) respectively in 0.6 mL of CD_2Cl_2 were measured at 20 °C, 600 MHz.

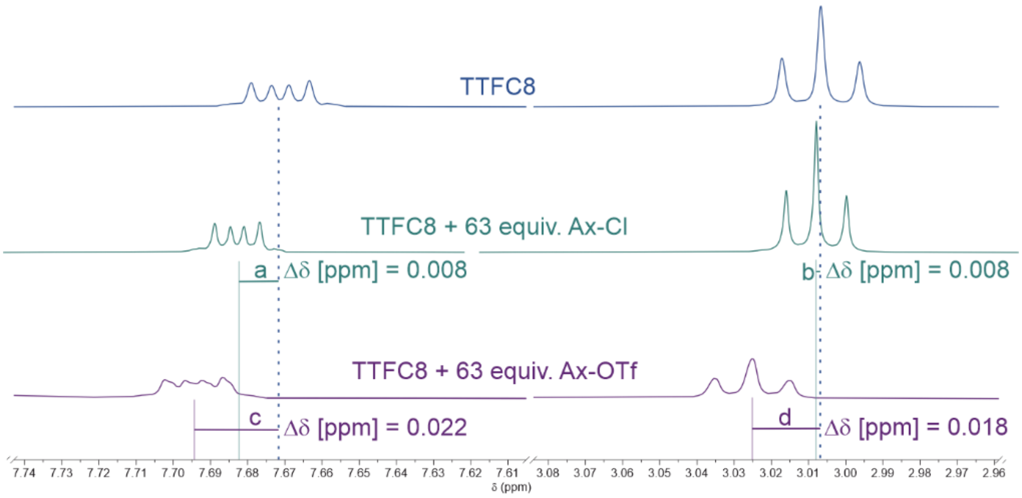

*Figure S 13: Investigation of the binding behavior of SCAs chloride and triflate: the signals of macrocycle **TTFC8** shift only insignificantly upon addition of 63 equiv. of **A1-Cl** or **A1-OTf** indicating, that no or only rather weak binding occurs. The observed shifts can be attributed to differences in the polarity of the concentrated axle solutions. Representative unobstructed signals were chosen for clearity.*

Section S5: Additional Computational results

Table S1: Experimental and theoretical ΔG_a values with respect to the involved anion (X) depending on the employed functional obtained using the SMD[10] solvent model and the def2-TZVP[11] level, respecitvely. All data are given in kJ/mol.

X	Cl^-	OTs^-	OTf^-	BF_4^-	NTf_2^-	PF_6^-	$BArF_{24}^-$	pf^-
$\Delta G_{a,exp.}$	---[1]	---[2]	---[1]	-8.2	-24.2	-25.7[3]	-32.2[3]	-32.2
M06-2X	-7.9	0.8	-13.0	-25.5	-23.6	-29.3	-30.1	-33.3
PBE0-D3(BJ)	-25.6	-29.9	-40.5	-48.7	-48.2	-56.7	-53.1	-55.8
ωB97X-D3	-38.2	-41.8	-49.6	-54.6	-53.3	-66.1	-60.4	-63.6

[1]*close to zero and, hence, not accurately determinable,* [2]*not measured,* [3]*from literature*[5]

Table S2: Theoretical ΔG_a values for the interaction with an anion (X) obtained SMD/M06-2X/def2-TZVP[12] level. All data are given in kJ/mol.

X	Cl^-	OTs^-	OTf^-	BF_4^-	NTf_2^-	PF_6^-	$BArF_{24}^-$	pf^-
**A1**	-56.8	-5.2	-11.0	-14.5	4.0	-30.0	19.9	26.8
**A1^s**	-58.4	-6.0	-7.0	-13.2	8.1	-38.5	27.7	35.2
**A1@TTFC8**	-15.7	14.5	12.5	21.6	29.5	19.8	38.8	42.4
**A1^s@TTFC8**	-19.5	8.6	-5.1	10.8	29.0	4.3	43.0	46.9

Table S3: Theoretical ΔG_a values for the interaction with an anion (X) obtained SMD/PBE0-D3(BJ)/def2-TZVP[13-15] level. All data are given in kJ/mol.

X	Cl^-	OTs^-	OTf^-	BF_4^-	NTf_2^-	PF_6^-	$BArF_{24}^-$	pf^-
**A1**	-62.8	-9.2	-14.5	-20.5	-11.4	-38.6	-5.9	10.3
**A1^s**	-61.7	-8.5	-9.4	-19.2	-5.3	-44.9	10.1	19.4
**A1@TTFC8**	-12.9	9.6	12.8	14.5	17.6	7.1	16.6	30.1
**A1^s@TTFC8**	-14.8	-3.6	-4.5	3.2	12.3	-8.7	16.3	20.4

Table S4: Theoretical ΔG_a values for the interaction with an anion (X) obtained SMD/wB97X-D3/def2-TZVP[16] level. All data are given in kJ/mol.

X	Cl^-	OTs^-	OTf^-	BF_4^-	NTf_2^-	PF_6^-	$BArF_{24}^-$	pf^-
**A1**	-58.4	-8.3	-17.7	-20.6	-12.5	-39.1	-8.3	10.2
**A1^s**	-59.0	-11.0	-10.3	-18.6	-5.5	-49.3	9.2	23.5
**A1@TTFC8**	-14.0	8.3	11.5	12.4	15.6	1.9	13.9	29.1
**A1^s@TTFC8**	-17.3	-4.8	-6.5	-1.23	10.0	-15.1	15.2	19.9

*Table S5: ΔG_{therm} contributions to ΔG_a for **A1@TTFC8** and **A1^s@TTFC8** obtained using the single-point Hessian approach.[17] All data are given in kJ/mol.*

X	Cl^-	OTs^-	OTf^-	BF_4^-	NTf_2^-	PF_6^-	$BArF_{24}^-$	pf^-
**A1@TTFC8**	77.4	79.7	77.2	85.6	83.6	82.0	88.1	80.9
**A1^s@TTFC8**	77.6	80.8	79.1	83.9	81.8	80.4	88.6	82.4

*Table S6: $\Delta G^{penalty}$ of **TTFC8** and **A1**-X obtained by comparing the free structures and the structures inside **A1@TTFC8** at the SMD/M06-2X/def2-TZVP level. The sum of the two values results in $\Delta G_a^{penalty}$(**A1@TTFC8**). All data are given in kJ/mol.*

X	Cl^-	OTs^-	OTf^-	BF_4^-	NTf_2^-	PF_6^-	$BArF_{24}^-$	pf^-
**TTFC8**	8.11	4.46	6.56	6.75	5.34	5.58	6.14	6.72
**A1**-X	49.88	52.68	44.98	37.81	37.35	28.64	39.59	29.50
**$DG_a^{penalty}$**	58.00	57.14	51.54	44.56	42.68	34.22	45.73	36.22

*Table S7: $\Delta G^{penalty}$ of **TTFC8** and **A1^s**-X obtained by comparing the free structures and the structures inside **A1^s@TTFC8** at the SMD/M06-2X/def2-TZVP level. The sum of the two values results in $\Delta G_a^{penalty}$(**A1^s@TTFC8**). All data are given in kJ/mol.*

X	Cl^-	OTs^-	OTf^-	BF_4^-	NTf_2^-	PF_6^-	$BArF_{24}^-$	pf^-
**TTFC8**	29.61	26.19	26.97	26.23	26.36	25.59	23.63	26.48
**A1^s**-X	60.35	80.35	50.16	37.01	49.37	37.13	31.50	31.50
**$DG_a^{penalty}$**	89.96	106.54	77.12	63.25	75.73	62.72	55.13	57.98

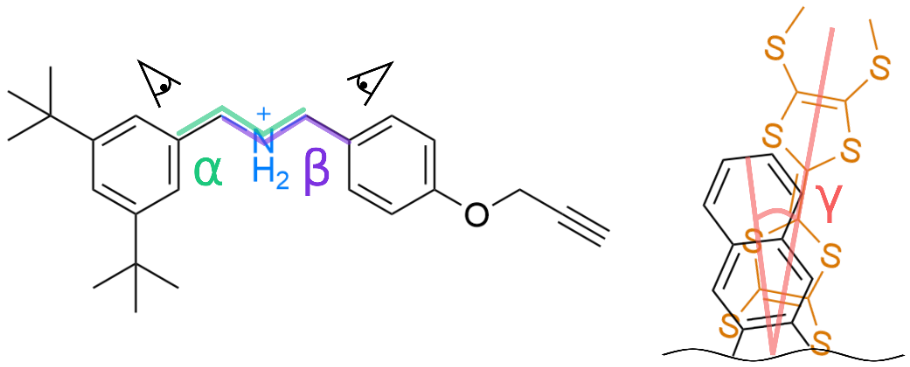

Figure S 14: Depiction of structural parameters (torsion angles) used to describe conformations in the following. α and β are used for the axle, γ is used for describing the macrocycle.

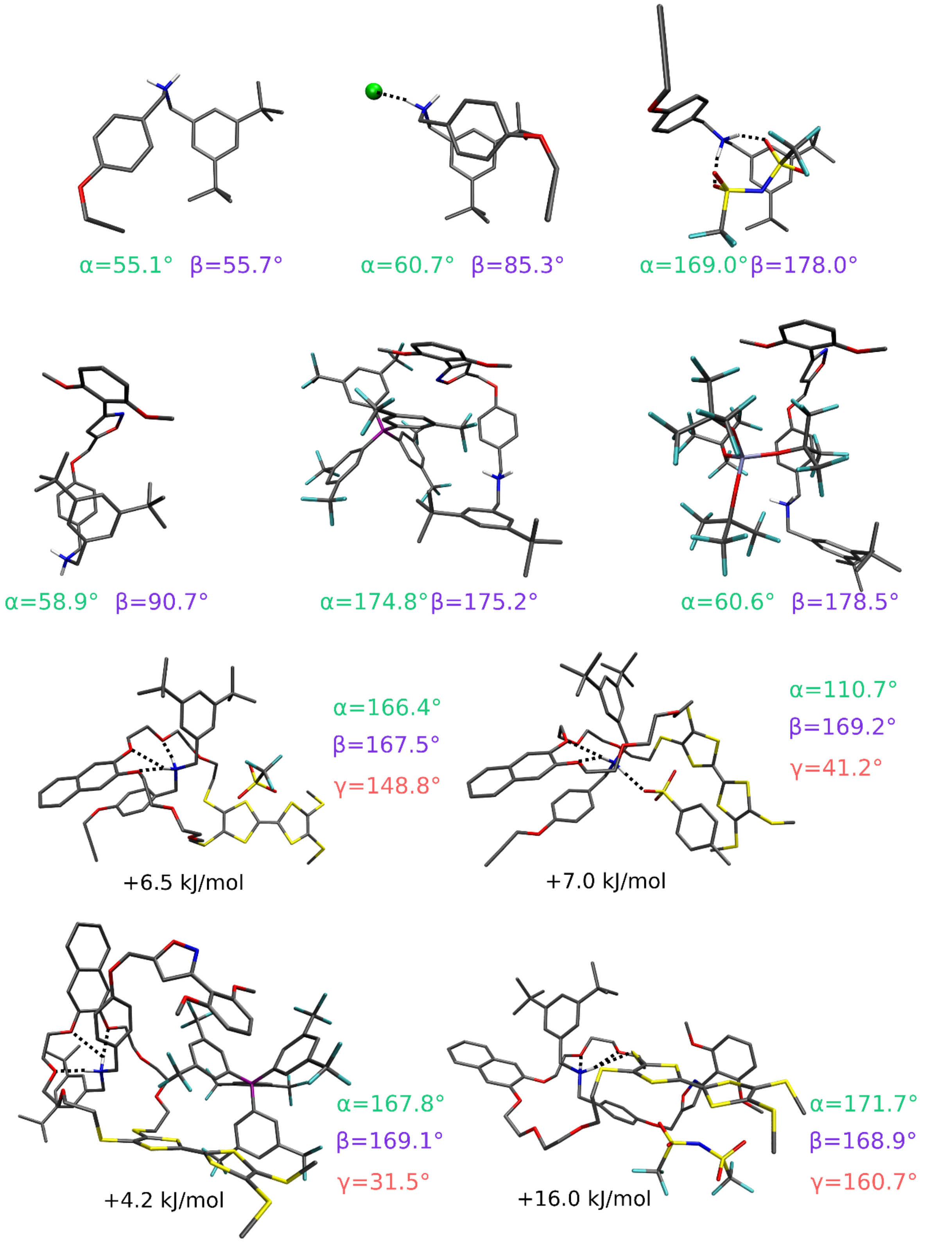

Figure S 15: A selection of conformations of ***A1***, ***A1^{s}***, ***A1@TTFC8***, *and* ***A1^{s}@TTFC8*** *(top to bottom) subject to various counterions. For the former two, the most stable conformations are shown, respectively. For the latter two, the differences in association free energy to the most stable conformation are given below the structures for comparison. Hydrogen atoms (except for those located at the ammonium centre) are omitted for clarity. Hydrogen bonds are illustrated using dashed lines. Next to each structure the parameters outlined in Figure S 14 are provided. Colour code: C-grey, O-red, N-blue, S-yellow, F-cyan, B-purple, Al-iceblue.*

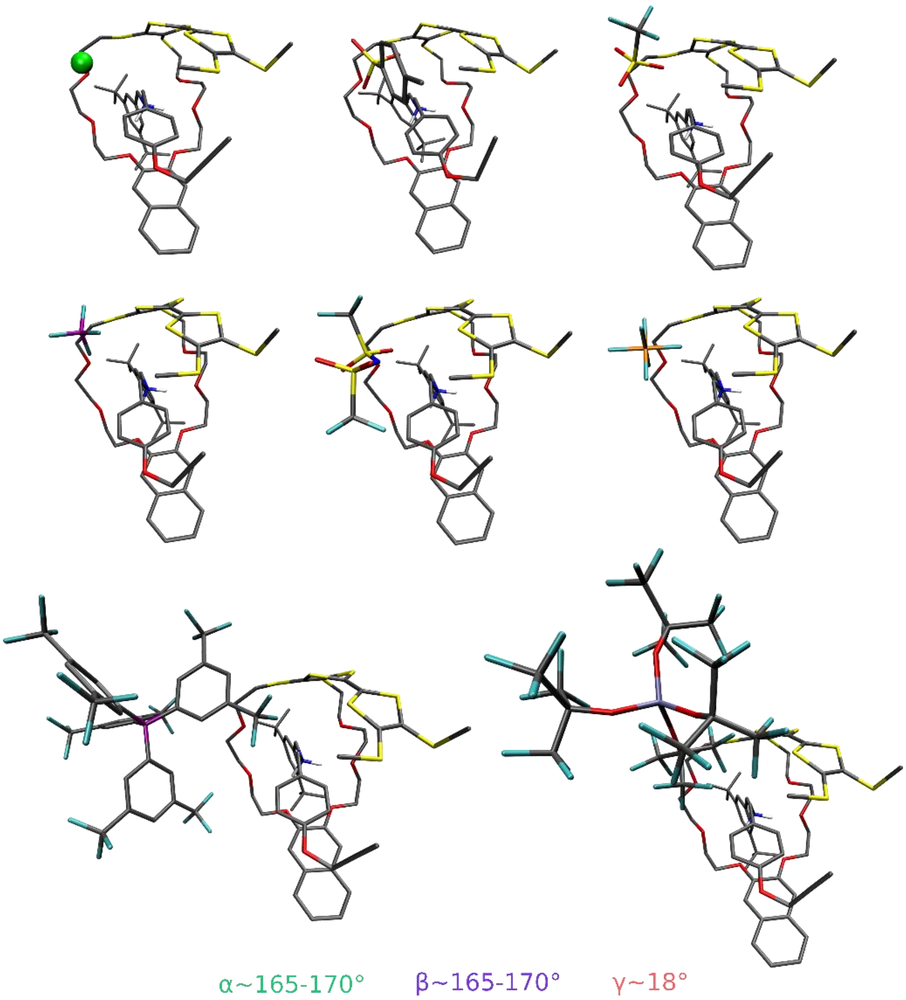

*Figure S 16: Most stable conformations of **A1@TTFC8** depending on the counterion from Cl^- (top left) to pf^- (bottom right). Phosphorus is depicted in orange, all other colours are in accordance with Figure S 15. The range of structural parameters according to Figure S 14 is given at the bottom.*

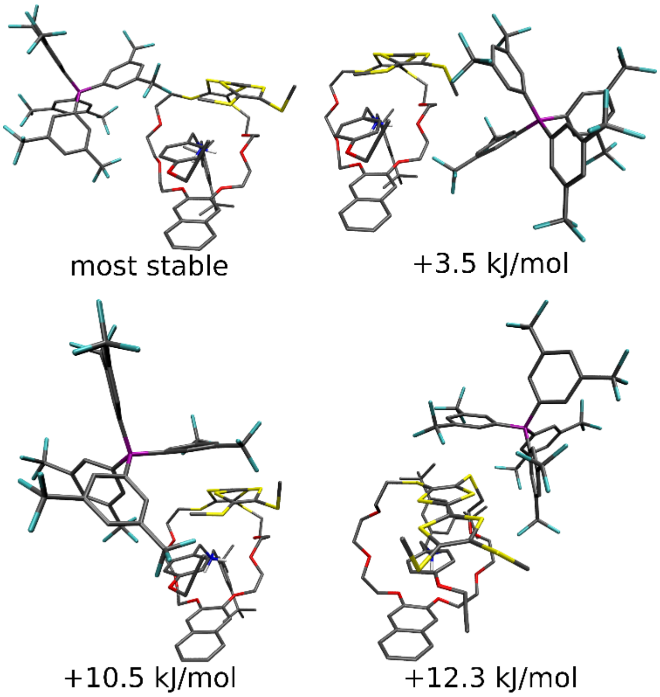

*Figure S 17: Relative stabilities (given below the structures) of **A1@TTFC8** in the presence of $BArF24^-$ at different positions. Colour code is in accordance with Figure S 15.*

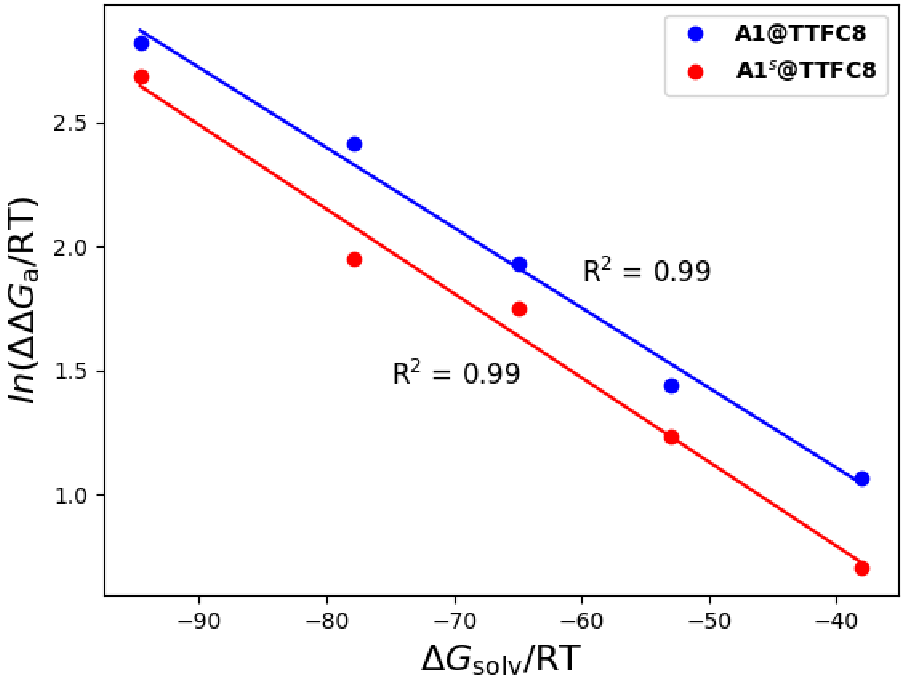

Figure S 18: Logarithmic plot of $\Delta\Delta G_a$ depending on solvation free energies ΔG_{solv} of the respective anion with coefficients of determination supporting the exponential behaviour of ΔG_a with respect to ΔG_{solv}. To highlight the observed trend, Cl^-, BF_4^-, and PF_6^- were omitted as these were most prone to systematical errors due to the lack of explicit solvent molecules in the calculations. All data were obtained at the SMD/M06-2X/def2-TZVP level of theory.

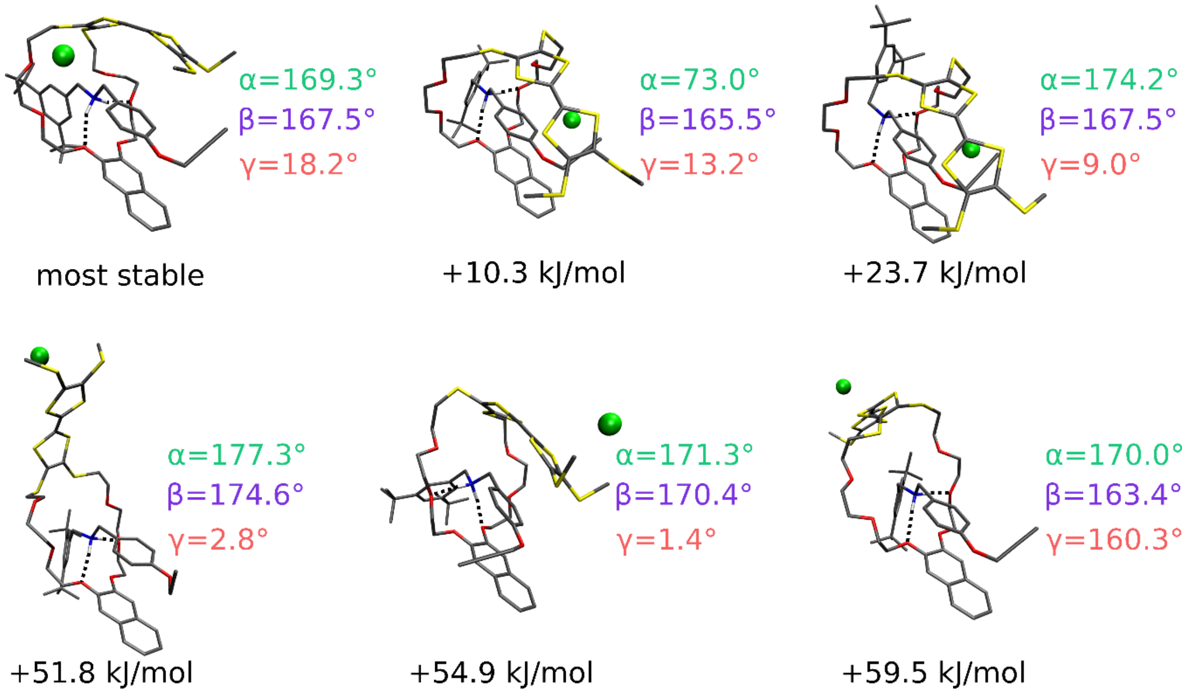

*Figure S 19: Chosen conformations of **A1@TTFC8** in the presence of Cl^- after the CREST run depicted after subsequent re-optimisation. Colours are in accordance with Figure S 15. Structural parameters according to Figure S 14 are given next to each structure.*

*Table S8: FMO-EDA[18,19] results obtained at the ωB97X-D3/cc-pVDZ[20] level for all **A1-X** combinations. E_{tot} is the sum of all the other contributions. See main text for the remaining quantities.*

X	E_{ES}	E_X	E_{CT}	E_{Disp}	E_{tot}
Cl^-	-129.9	28.8	-22.2	-6.6	-130.0
OTs^-	-126.5	44.8	-25.8	-28.9	-136.5
OTf^-	-103.2	25.3	-18.9	-19.5	-116.2
BF_4^-	-106.3	17.2	-16.6	-10.7	-116.3
NTf_2^-	-102.8	31.8	-19.8	-26.5	-117.3
PF_6^-	-85.5	15.3	-13.7	-15.3	-99.3
$BArF_{24}^-$	-58.2	34.4	-20.2	-46.2	-90.1
pf^-	-54.7	20.9	-15.8	-29.2	-78.8

*Table S9: FMO-EDA results obtained at the ωB97X-D3/cc-pVDZ level for all **A1-TTFC8** interactions within **A1@TTFC8** in the presence of an anion. E_{tot} is the sum of all the other contributions. See main text for the remaining quantities.*

X	E_{ES}	E_X	E_{CT}	E_{Disp}	E_{tot}
Cl^-	-74.9	91.8	-34.1	-95.3	-112.6
OTs^-	-73.5	86.0	-32.3	-90.2	-110.0
OTf^-	-79.7	91.4	-33.8	-94.1	-116.2
BF_4^-	-81.0	92.9	-34.0	-95.3	-117.4
NTf_2^-	-80.8	90.9	-33.5	-93.8	-117.2
PF_6^-	-80.1	92.5	-34.2	-94.6	-116.4
$BArF_{24}^-$	-84.2	90.0	-33.8	-93.2	-121.2
pf^-	-84.2	90.0	-33.8	-93.2	-121.2

Section S6: Theoretical Calculations of **A2@BC7**

The procedure outlined in section 2.2 and the Materials and Methods section in the main text was repeated for a smaller and less complex supramolecule **A2@BC7** (Figure 1c).

Table S10: Experimental and theoretical ΔG_a values with respect to the involved anion (X) depending on the employed functional obtained using the SMD solvent model and the def2-TZVP level, respecitvely. All data are given in kJ/mol.

X	Cl^-	OTs^-	OTf^-	BF_4^-	NTf_2^-	PF_6^-	$BArF_{24}^-$	pf^-
M06-2X	12.7	-0.7	-14.6	-9.5	-20.7	-27.6	-39.8	-37.9
PBE0-D3(BJ)	2.9	-9.0	-26.3	-14.7	-31.4	-40.0	-60.1	-54.9
ωB97X-D3	3.3	-13.9	-31.8	-31.3	-33.1	-43.5	-63.8	-56.9

The numbers in Table S10 may be compared to the literature value for this association reaction with PF_6^- of -34.6 kJ/mol.[5] Note that $BArF_{24}^-$ seems to be somewhat overstabilised. Just like for **A1@TTFC8** and **A1^s@TTFC8**, the conformational analysis yields that the most stable conformation (Figure S20) is retained irrespective of the involved anion.

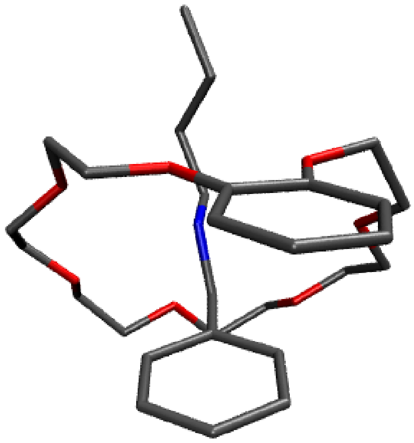

*Figure S 20: Most stable conformation of **A2@BC7**.*

*Table S11: Results obtained from the analysis of Equation 3 for **A2@BC7** computed at the SMD/M06-2X/def2-TZVP level. The ΔG_a^{opt} value is given in kJ/mol. k1 and k2 are dimensionless. Note that $BArF_{24}^-$ had to be omitted from the analysis as otherwise no convergence of the determination coefficient could be achieved.*

ΔG_a^{opt}	k_1	k_2
-42.78	0.687	0.033

*Table S12: FMO-EDA results obtained at the ωB97X-D3/cc-pVDZ level for all **A2**-X combinations. E_{tot} is the sum of all the other contributions. See main text for the remaining quantities.*

X	pE_{ES}	E_{ES}	E_X	E_{CT}	E_{Disp}	E_{tot}
Cl^-	0.82	-130.5	26.9	-21.2	-7.1	-131.9
OTs^-	0.77	-122.8	29.7	-21.0	-16.5	-130.6
OTf^-	0.79	-109.4	22.1	-17.5	-11.4	-116.2
BF_4^-	0.83	-102.1	11.8	-13.0	-7.7	-110.9

NTf_2^-	0.76	-104.2	23.4	-16.0	-16.4	-113.2
PF_6^-	0.82	-88.3	10.7	-11.5	-8.4	-97.6
$BArF_{24}^-$	0.59	-57.8	19.0	-11.8	-28.6	-79.2
pf⁻	0.75	-55.3	5.8	-8.3	-10.4	-68.3

*Table S13: FMO-EDA results obtained at the ωB97X-D3/cc-pVDZ level for all **A2-BC7** interactions within **A2@BC7** in the presence of an anion. E_{tot} is the sum of all the other contributions. See main text for the remaining quantities.*

X	pE_{ES}	E_{ES}	E_X	E_{CT}	E_{Disp}	E_{tot}
Cl^-	0.48	-75.4	65.2	-29.3	-52.5	-91.9
OTs^-	0.48	-76.9	62.8	-30.2	-54.4	-98.8
OTf^-	0.48	-74.6	56.7	-28.6	-50.7	-97.3
BF_4^-	0.48	-75.4	58.1	-28.8	-52.0	-98.1
NTf_2^-	0.49	-75.9	60.6	-28.7	-51.3	-95.3
PF_6^-	0.47	-76.9	63.4	-30.1	-55.3	-99.0
$BArF_{24}^-$	0.5	-78.6	58.9	-28.9	-50.4	-99.0
pf^-	0.49	-78.8	60.7	-29.0	-51.6	-98.8

The differences among the various **A2**-X combinations are not as pronounced as for **A1-**X. The electrostatic contribution is the most significant one for both **A2**-X and **A2**-**BC7**, while the quantum-mechanical effects remain much smaller. This can be attributed to the smaller size of the molecule on the one hand, on the other it is also in line with its overall simplicity in comparison to **A1@TTFC8** or **A1^s@TTFC8**.

References

1 N. Svenstrup, K. M. Rasmussen, T. K. Hansen and J. Becher, *Synthesis*, 1994, **1994**, 809.

2 H. V. Schröder, H. Hupatz, A. J. Achazi, S. Sobottka, B. Sarkar, B. Paulus and C. A. Schalley, *Chem. Eur. J.*, 2017, **23**, 2960.

3 Z.-J. Zhang, H.-Y. Zhang, H. Wang and Y. Liu, *Angew. Chem. Int. Ed.*, 2011, **50**, 10834.

4 H. V. Schröder, S. Sobottka, M. Nößler, H. Hupatz, M. Gaedke, B. Sarkar and C. A. Schalley, *Chem. Sci.*, 2017, **8**, 6300-6306.

5 Hupatz, H.; Gaedke, M.; Schröder, H. V.; Beerhues, J.; Valkonen, A.; Klautzsch, F.; Müller, S.; Witte, F.; Rissanen, K.; Sarkar, B.; Schalley, C. A. *Beilstein J. Org. Chem.* **2020,** *16,* 2576–2588.

6 M. Gaedke , F. Witte , J. Anhäuser , H. Hupatz , H. V. Schröder , A. Valkonen , K. Rissanen , A. Lützen , B. Paulus and C. A. Schalley , Chiroptical inversion of a planar chiral redox-switchable rotaxane, *Chem. Sci.*, 2019, **10** , 10003.

7 Jones, J. W.; Gibson, H. W. *J. Am. Chem. Soc.*, **2003**, 125, 7001–7004.

8 Gibson, H. W.; Jones, J. W.; Zakharov, L. N.; Rheingold, A. L.; Slebodnick, C. *Chem. – Eur. J.,* **2011**, 17, 3192–3206.

9 Hansen, T. K.; Becher, J.; Jørgensen, T.; Varma, K. S.; Khedekar, R.; Cava, M. P. *Org. Synth.* **1996**, 73, 270.

10 A. V. Marenich, C. J. Cramer, D. G. Truhlar, *J. Phys. Chem. B* **2009**, 113, 6378–6396.

11 F. Weigend, R. Ahlrichs, *Phys. Chem. Chem. Phys.* **2005**, 7, 3297.

12 Y. Zhao and D. G. Truhlar, *Theor. Chem. Acc.* **2007**, 120, 215-241.

13 J. P. Perdew, M. Ernzerhof, K. Burke, *J. Chem. Phy*s. **1996**, 105, 9982–9985.

14 S. Grimme, J. Antony, S. Ehrlich, H. Krieg, *J. Chem. Phy*s. **2010**, 132, 154104.

15 S. Grimme, S. Ehrlich, L. Goerigk, *J. Comput*. *Chem*. **2011**, 32, 1456–1465.

16 J.-D. Chai, M. Head-Gordon, *J. Chem. Phys.* **2008**, 128, 084106.

17 S. Spicher, S. Grimme, *Chem. Theory Comput.* **2021**, 17, 1701−1714.

18 K. Kitaura, E. Ikeo, T. Asada, T. Nakano, M. Uebayasi, *Chem. Phys. Lett.* **1999**, 313, 701–706.

19 K. Kitaura, K. Morokuma, *Int. J. Quantum Chem.* **1976**, 10, 325–340.

20 T. H. Dunning, Thomas H., *J. Chem. Phys.* **1989**, 90, 1007–1023.
